# Engineered bilayer hydrogel with spatiotemporal drug and oxygen delivery for diabetic wound microenvironment reprogramming

**DOI:** 10.1093/rb/rbaf134

**Published:** 2025-12-27

**Authors:** Huaping Li, Quan Chen, Bihua Liang, Huiyan Deng, Chao Bi, Liqian Peng, Jiaoquan Chen, Shanshan Ou, Luoyu Zhang, Ziyan Chen, Huilan Zhu

**Affiliations:** Guangzhou Dermatology Hospital, Institute of Dermatology, Guangzhou Medical University, Guangzhou, Guangdong 510030, China; Guangzhou Dermatology Hospital, Institute of Dermatology, Guangzhou Medical University, Guangzhou, Guangdong 510030, China; Guangzhou Dermatology Hospital, Institute of Dermatology, Guangzhou Medical University, Guangzhou, Guangdong 510030, China; Guangzhou Dermatology Hospital, Institute of Dermatology, Guangzhou Medical University, Guangzhou, Guangdong 510030, China; Guangzhou Dermatology Hospital, Institute of Dermatology, Guangzhou Medical University, Guangzhou, Guangdong 510030, China; Guangzhou Dermatology Hospital, Institute of Dermatology, Guangzhou Medical University, Guangzhou, Guangdong 510030, China; Guangzhou Dermatology Hospital, Institute of Dermatology, Guangzhou Medical University, Guangzhou, Guangdong 510030, China; Guangzhou Dermatology Hospital, Institute of Dermatology, Guangzhou Medical University, Guangzhou, Guangdong 510030, China; Guangzhou Dermatology Hospital, Institute of Dermatology, Guangzhou Medical University, Guangzhou, Guangdong 510030, China; Guangzhou Dermatology Hospital, Institute of Dermatology, Guangzhou Medical University, Guangzhou, Guangdong 510030, China; Guangzhou Dermatology Hospital, Institute of Dermatology, Guangzhou Medical University, Guangzhou, Guangdong 510030, China

**Keywords:** anti-fouling, boronic ester bond, oxygen release, anti-inflammatory, diabetic wound healing

## Abstract

The impaired healing of diabetic wounds primarily stems from persistent inflammation, a hypoxic microenvironment, and heightened susceptibility to infection. However, most existing studies focus on simple functional stacking, rather than aligning with the dynamic pathological repair process, which hinders the maximization of therapeutic efficacy of the repair materials. In this study, an intelligently responsive, bilayer anti-fouling nanocomposite hydrogel (Ca@Q-E@SGH) was developed for spatiotemporally synergistic therapy via spatiotemporal drug and oxygen delivery strategies. Its core component (Ca@Q-E) consists of calcium peroxide encapsulated by phenylboronic acid-modified quaternary ammonium chitosan, with epigallocatechin gallate (EGCG) linked via boronate esters. This dynamic bond enables ROS/glucose-responsive EGCG release to reprogram macrophages from the M1 to M2 phenotype, mitigating early-stage inflammation. As the matrix degrades, sustained oxygen is released from CaO_2_, supporting vascularization during tissue remodeling. Furthermore, the bilayer hydrogel structure is designed to provide multiple protective functions: the lower layer rapidly crosslinks to encapsulate the functional nanoparticles, while the upper layer forms a highly hydrophilic anti-fouling coating that effectively prevents pathogen adhesion. Collectively, this integrated platform combines intelligent microenvironment-responsive drug release for antibacterial and anti-inflammatory effects, followed by sequential oxygen delivery aligned with the wound healing stages, along with physical anti-fouling protection. As a result, the treated wounds achieved a remarkable closure rate of 99.1% by day 14. This study presents a comprehensive strategy for diabetic wound management by seamlessly integrating smart anti-inflammatory action, prolonged oxygen supply, and efficient anti-fouling capacity into a single coordinated platform.

## Graphical abstract

**Figure:**
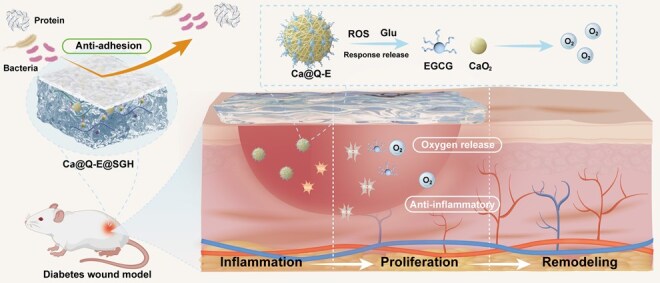

## Introduction

Diabetic patients persistently face the risks of chronic inflammation and refractory wound healing, which significantly increase the rates of non-traumatic amputations and global mortality [1, 2]. The four sequential yet overlapping stages of diabetic wound healing (hemostasis, inflammation, proliferation, and remodeling) are frequently disrupted [3, 4]. The persistent hyperglycemic environment promotes excessive protein glycation and accumulation of advanced glycation end products (AGEs), while simultaneously stimulating immune cells to overproduce reactive oxygen species (ROS), resulting in excessive oxidative stress [5]. The synergistic effects of factors such as hyperglycemia, advanced glycation end products (AGEs), reactive oxygen species (ROS), and hypoxia can lead to mitochondrial dysfunction. The persistent accumulation of dysfunctional mitochondria and the subsequent release of mitochondrial damage-associated molecular patterns (mtDAMPs) result in the over-activation of inflammatory signaling pathways, including the NLRP3 inflammasome and the cGAS-STING axis, ultimately contributing to the establishment of refractory chronic inflammation [6]. Furthermore, under diabetic conditions, the phenotypic transition from M1 to M2 macrophages is impaired, compromising macrophage plasticity. Persistent oxidative stress activates signaling pathways such as NF-κB, MAPK/ERK [7], and PI3K/AKT [8], which not only sustain the activation of pro-inflammatory M1 macrophages—leading to the excessive production of pro-inflammatory cytokines like TNF-α and IL-1β—but also suppress the shift toward reparative M2 macrophages. The latter is essential for resolving inflammation and promoting tissue repair through the secretion of factors such as TGF-β and VEGF. The impaired vascular system leads to inadequate local tissue perfusion (hypoxia), which, combined with pre-existing compromised blood supply and accumulation of inflammatory mediators, makes it difficult to achieve complete healing of diabetic wounds [9]. Consequently, the development of novel functional materials capable of actively intervening in and improving the complex diabetic wound microenvironment (e.g., hypoxia, oxidative stress, and persistent inflammation) is crucial for achieving effective treatment of diabetic wounds [10, 11].

Excessive reactive oxygen species (ROS) are a key factor impeding diabetic wound healing. They can induce irreversible biomolecular damage, exacerbate oxidative stress and inflammatory responses, and hinder the transition of macrophages from pro-inflammatory M1 to anti-inflammatory M2 phenotypes, ultimately leading to chronic inflammation [12, 13]. Additionally, uncontrolled ROS accumulation severely suppresses the proliferation and differentiation capabilities of endogenous stem cells, functional cells, and growth factors in wound tissue, significantly impairing tissue regeneration potential [14]. Current research widely employs metal nanozymes to scavenge free radicals and promote wound repair. These nanozymes function by mimicking natural antioxidant enzymes such as catalase (CAT), glutathione peroxidase (GPx) and superoxide dismutase (SOD) [15, 16]. However, nanozymes face challenges such as rapid *in vivo* metabolism, uncontrollable catalytic rates, and potential immune responses or metal ion accumulation toxicity upon prolonged exposure [17]. In contrast, natural polyphenols (e.g., curcumin Cur, tea polyphenols TP, epigallocatechin gallate [EGCG]) exhibit free radical scavenging and anti-inflammatory properties, but their applications are limited by low bioavailability and poor stability [18, 19]. An efficient drug delivery system is crucial to overcoming these limitations: metal-organic frameworks (MOFs) serve as ideal carriers due to their high porosity, while polymeric micelles and liposomes offer high drug-loading capacity. Nevertheless, most of the existing nano-delivery systems lack the ability for stimulus-responsive release and the precise controlled release of drugs [20]. Thus, developing intelligent and controllable delivery strategies holds significant importance for diabetic wound therapy.

In diabetic wounds, impaired angiogenesis leads to chronic oxygen deprivation, which hinders the repair process by suppressing vascularization, epithelialization, and extracellular matrix (ECM) regeneration [21]. Conventional wound dressings may further block oxygen diffusion from the air to the wound tissue. Therefore, designing materials capable of providing sustained and sufficient oxygen to hypoxic areas remains a significant challenge [22]. Oxygen-releasing systems enhance wound healing under hypoxic conditions by enabling continuous oxygen supply. Among various oxygen-generating compounds (e.g., 2Na_2_CO_3_·3H_2_O_2_, CaO_2_, MgO_2_, H_2_O_2_) [23], CaO_2_ is a widely used solid oxygen donor. It is renowned for its prolonged oxygen release and the ease of obtaining high-purity CaO_2_ [24], making it one of the most reliable oxygen-releasing materials for biomedical applications. Notably, the released Ca^2+^ can accelerate diabetic wound healing by promoting angiogenesis, enhancing re-epithelialization, and facilitating collagen deposition and tissue remodeling [25]. However, the direct application of CaO_2_ to wounds carries potential risks, such as excessive H_2_O_2_ accumulation, which may trigger undesirable inflammatory responses. This necessitates a highly precise release mechanism to meet the varying demands of different wound healing stages [26]. However, most existing strategies rely on the simple superposition of functions (such as anti-inflammation, antibacterial activity and oxygen supply), which fails to align with the dynamic demands of the diabetic wound healing process [27]. Therefore, we designed an integrated multifunctional material that programmatically couples these actions: it intelligently responds to the wound microenvironment to enable the sequential release of anti-inflammatory agents, antibacterial components, and oxygen, thereby multi-dimensionally rectifying the pathological microenvironment and promoting tissue regeneration.

**Figure 1:**
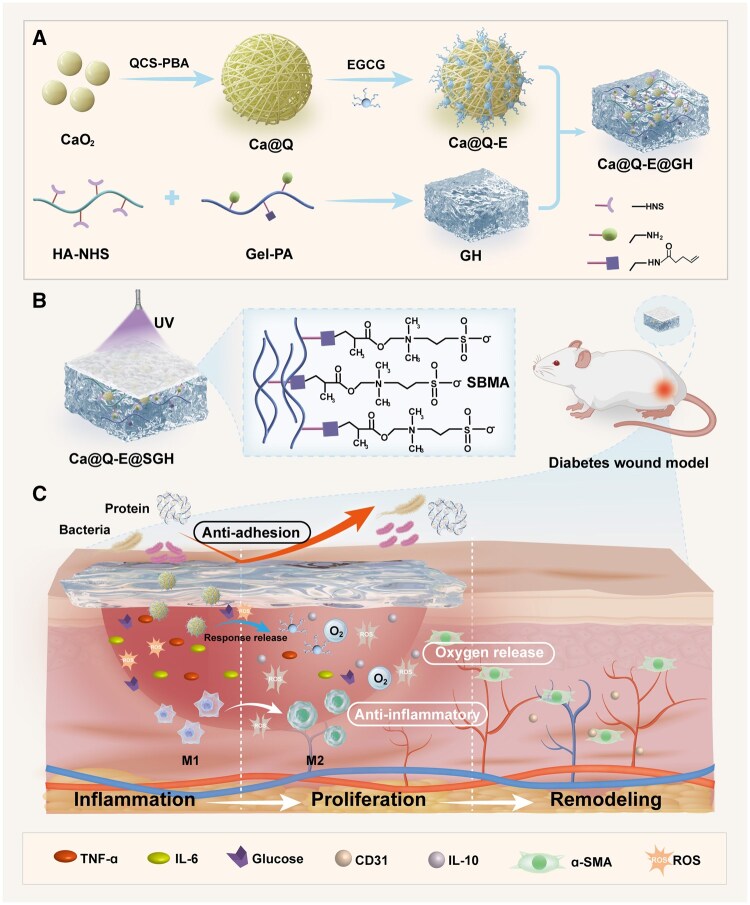
The preparation flowchart and therapeutic mechanism diagram of multiple functional synergy advanced smart hydrogel. (**A**) Preparation of drug-loaded hydrogel. (**B**) Preparation of anti-fouling coating hydrogel. (**C**) The Ca@Q-E@SGH hydrogel induces macrophage polarization through anti-fouling, anti-inflammatory and oxygen-release synergistic effects, accelerates angiogenesis, and promotes the healing of diabetic wounds.

To address these challenges, this study designed and developed an intelligent controlled-release system integrating anti-inflammatory, oxygen-releasing, and anti-fouling functions (Figure 1). The system employs CaO_2_ as the core oxygen-generating component, which is encapsulated by phenylboronic acid-modified quaternized chitosan (QCS-PBA) to effectively suppress the burst release of CaO_2_. This structure further utilizes dynamic borate ester bonds formed between phenylboronic acid groups and the phenolic hydroxyl groups of EGCG, enabling dual-responsive (ROS and glucose) precise release of EGCG (Ca@Q-E). This nanosystem with both intelligent antibacterial, anti-inflammatory and sustained oxygen-releasing properties. To tackle the issue of bacterial adhesion and contamination in diabetic wounds, a dual-layer anti-fouling hydrogel was concurrently designed. The lower layer consists of N-hydroxysuccinimide (NHS)-modified hyaluronic acid (HA-NHS) and 4-pentenoic acid (PA)-modified gelatin (Gel-PA), crosslinked via rapid NHS ester bond formation, and serves as a carrier for the Ca@Q-E nanoparticles. Subsequently, under UV irradiation, the double bonds on PA undergo polymerization with sulfobetaine methacrylate (SBMA), forming an anti-fouling coating (SGH) on the hydrogel surface. The synergistic design of this intelligent nanosystem (Ca@Q-E) and the anti-fouling bilayer hydrogel demonstrates significant potential in achieving precise inflammation regulation, sustained oxygenation, and long-term anti-fouling/antibacterial protection, providing an innovative strategy for the effective management of diabetic chronic wounds.

## Materials and methods 

### Materials

Calcium chloride dihydrate (CaCl_2_·2H_2_O, 99%), Sodium hydroxide (NaOH, 95%), 2-Formylphenylboronic acid (2-FPBA, 98%), Chitosan quaternary ammonium salt (QCS, degree of substitution 92%), Epigallocatechin gallate (EGCG, 95%), Hyaluronic acid (HA, Mw = 100 000 - 200 000), N-(3-Dimethylaminopropyl)-N'-ethylcarbodiimide hydrochloride (EDC, 98.5%), Gelatin (Gel, source: Cow Bone), 4-Pentenoic acid (4-PA, 98%), [2-(Methacryloyloxy) ethyl] dimethyl-(3-sulfopropyl) ammonium hydroxide (SBMA, 98%), N, N'-Methylenebis(acrylamide) (MBAA, 97%) and 2-Hydroxy-4′-(2-hydroxyethoxy)-2-methylpropiophenone (I2959, 98%) were purchased from Shanghai Aladdin Biochemical Technology Co., Ltd N-Hydroxy succinimide (NHS) were purchased from Shanghai Yuanye Technology Co.

### Synthesis and characterization of Ca@Q-E nanoparticles

The synthesis of CaO_2_ nanoparticles was initiated by dissolving 53 g of CaCl_2_·2H_2_O in 200 mL of ultrapure water, followed by ultrasonic homogenization for 15 min to ensure complete dissolution. Then, 80 mL of 1 M sodium hydroxide solution was added to the mixture under stirring. After thorough mixing, 100 mL of H_2_O_2_ (7.7%) was slowly added dropwise, followed by continuous stirring at room temperature (RT) for 2 h. During this process, gradual particle precipitation was observed, forming a white suspension. The product was collected by centrifugation at 10 000 rpm, washed, and dried at 80°C for 2 h [26].

Synthesis of QCS-PBA: 1 g of QCS was dissolved in 100 ml of deionized water, followed by the addition of 200 mg of 2-FPBA. Subsequently, 2.14 g EDC and 0.79 g NHS were introduced, and the reaction proceeded under stirring at RT for 12 h. The resulting product was purified using a 3.5 kDa molecular weight cutoff dialysis membrane for 3 days, followed by lyophilization to obtain the QCS-PBA conjugate. The successful loading of EGCG was confirmed by ultraviolet-visible (UV-Vis) spectrophotometry [28]. The structure of QCS-PBA was characterized by nuclear magnetic resonance hydrogen spectroscopy (^1^H-NMR) and Fourier transform infrared (FT-IR) spectroscopy, and the degree of substitution of PBA was calculated using formula (1):


(1) $$\text{PBA substitution degree (\%)}=(\frac{S_{1}/4}{S_{1}/4+S_{2}/2})\;*\;100\%$$

Among them, *S*_1_ represents the integral area of the proton on the PBA benzene ring, and *S*_2_ represents the integral area of the amine proton in the QCS.

Fabrication of Ca@Q-E nanoparticles: 100 mg of CaO_2_ nanoparticles were dispersed in 40 ml of a 2.5 mg/mL QCS-PBA solution and stirred for 3 h at RT. After centrifugation at 10 000 rpm and two washing cycles with deionized water, the particles were redispersed in 40 mL of water. Then, 20 mg of EGCG was added, and the mixture was stirred for an additional 3 h. The final Ca@Q-E nanocomposite was collected by centrifugation, washed and freeze-dried.

The CaO_2_, Ca@Q and Ca@Q-E nanoparticles were dispersed in an ethanol solution, and after 5 min of ultrasonic treatment, their morphologies were observed using transmission electron microscopy (TEM), and their particle size distributions were statistically analyzed. The surface charge properties of the nanoparticles were evaluated by measuring their zeta potentials using dynamic light scattering (DLS). The content of Ca^2+^ in Ca@Q-E nanoparticles was detected by inductively coupled plasma optical emission spectrometry (ICP-OES).

### Synthesis of Ca@Q-E@SGH hydrogel

Synthesis of HA-NHS: 1 g of HA was dissolved in 50 ml of DMSO in a conical flask. EDC and NHS were sequentially added with an EDC/NHS molar ratio of 1:1 and an NHS/COOH ratio of 4:1. After 24 h of reaction, the product was precipitated by adding acetone, followed by one ethanol wash. The final product was lyophilized and stored for future use [29].

Synthesis of Gel-PA: 5 g of gelatin was first swollen in 100 ml of cold water for 30 min, then completely dissolved by stirring in a 60°C water bath. EDC and NHS were added sequentially with 30 min of stirring, followed by addition of 1 g PA. The reaction proceeded for 12 h at room temperature under light-protected conditions. The product was then dialyzed using a 3500 kDa membrane for 3 days before freeze-drying. The successful synthesis of HA-NHS and Gel-PA was confirmed by ^1^H NMR spectroscopy and the degree of substitution of PA and NHS respectively according to Formula (2) and Formula (3) was calculated:


(2) $$\text{PA substitution degree (\%)}=(\frac{S_{1}/3}{S_{1}/3+S_{2}/2})\;*\;100\%$$

Among them, *S*_1_ represents the integral area of the proton on the vinyl group of PA, and *S*_2_ represents the integral area of the amine proton in the Gel.


(3) $$\text{NHS substitution degree (\%)}=(\frac{S_{1}/4}{S_{2}/3})\;*\;100\%$$

Among them, *S*_1_ represents the integral area of the NHS five-membered ring proton, and *S*_2_ represents the integral area in HA.

Fabrication of Ca@Q-E@SGH hydrogel: The Ca@Q-E@GH hydrogel layer was first prepared by thoroughly mixing 10 wt% Gel-PA and 5 wt% HA-NHS at 1:1 volume ratio, followed by incorporation of 0.1 wt% Ca@Q-E nanoparticles. The mixture was allowed to gel in a 15 mm diameter cylindrical mold. For the upper layer, 0.3% I2959 photoinitiator was added to 20 wt% SBMA solution, with 50 μL of 100 mg/mL MBAA crosslinker per 1 mL solution. 200 μL of this prepolymer solution was applied onto the Ca@Q-E@GH hydrogel surface and crosslinked under 405 nm UV light for 5 min to form the final Ca@Q-E@SGH hydrogel [30].

### Oxygen release test

The oxygen release amount of CaO_2_ nanoparticles was determined using a dissolved oxygen meter. One gram of CaO_2_ nanoparticles was weighed and added to 20 ml of distilled water. The dissolved oxygen meter was used to measure the oxygen release amount of CaO_2_ and Ca@Q-E nanoparticles at different time points under a constant temperature of 37°C [24].

### Mechanical property testing

The viscoelastic properties of the hydrogels were characterized using a rotational rheometer equipped with a parallel-plate geometry. All measurements were conducted at a physiological temperature of 37°C. The tests included the following frequency sweep (fixed strain of 1.0%, ranging from 0.1–100 rad/s) and strain amplitude sweep (fixed angular frequency of 10.0 rad/s, from 0.01% to 100%).

GH, SGH and Ca@Q-E@SGH hydrogels with a diameter of 15 mm were prepared using molds. The compression performance was tested (at 80% strain) using a universal testing machine (Sans, Shenzhen) at a speed of 3 mm/min, and the compression stress–strain curves and compression modulus were obtained.

### Anti-fouling test

The anti-protein experiment was conducted using the BCA method. 200 μL of 1 mg/mL bovine serum albumin (BSA) was added to the SGH and GH hydrogel. A 20 μL aliquot of the supernatant was collected following a 30-min incubation period and subsequently mixed with 200 μL of chromogenic substrate solution to initiate the colorimetric reaction. The BSA concentration in the supernatant was measured using a 562 nm enzyme detector, and the residual protein rates at the surface of GH and SGH were calculated according to formula (4) [31].


(4) $$\text{Protein residue ratio (\%)}=(1-\frac{C_{S}}{C_{0}})\;*\;100\%$$

Here, *C*_S_ and *C*_0_ represent the concentration of BSA in the sample supernatant and the initial concentration of BSA added, respectively.

The antibacterial adhesion experiment was conducted using crystal violet staining method. 200 μL of *Escherichia coli* (*E. coli*) and *Staphylococcus aureus* (*S. aureus*) (1 × 10^6^ CFU/mL) were dropped onto the surface of GH and SGH, and incubated for 1 h. Then, the adherent bacteria on the surface were subjected to sequential processing involving deionized water rinsing, staining with 1% crystal violet for 20 min, followed by additional deionized water washing steps. The residual crystal violet photos were taken and recorded. Subsequently, the remaining crystal violet on the surface was extracted with ethanol, and the OD value was measured using a microplate reader with a wavelength of 590 nm [32].

### Release performance

In 1 g of Ca@Q-E@SGH hydrogel, 5 mL of PBS (pH 7.4), 10 mM H_2_O_2_ and 10 mM Glu were added, respectively. To assess the release kinetics of EGCG, the samples were maintained at 37°C with periodic measurements taken at predetermined intervals. To determine the cumulative EGCG release, the optical density of the supernatant was quantified at approximately 280 nm using UV-Vis spectroscopy.

### Antibacterial property

*E. coli* and *S. aureus* were selected to test the antibacterial properties of SGH, Ca@Q-E@SGH and Ca@Q-E. 1 mL (1 × 10^8^ CFU/mL of bacterial solution was added to 0.5 g SGH, Ca@Q-E@SGH and 100 μg/mL of Ca@Q-E, respectively. The mixture was incubated in a 100 rpm, 37°C shaking incubator. 100 μL of bacterial solution was taken at different time points to measure the OD value and the bacterial growth curve was plotted. After incubation of the materials and the bacterial solution for 8 h, 100 μL of the bacterial solution was diluted 10^6^ times and used for plating on the plate. The plate was incubated in a 37°C incubator overnight. The formation of colonies in each group was recorded and the bacterial clearance rate was calculated according to formula (5):


(5) $$\mathrm{Bacterial}\;\mathrm{survival}\;\mathrm{ratio}\;(\%)=\frac{N_{\mathrm{hydrogel}}}{N_{\mathrm{blank}}}\times 100\%$$

The bacterial colony enumeration results are expressed as *N*_blank_ for the control group and *N*_hydrogel_ for the hydrogel-treated specimens.

SEM of bacterial: 0.5 g hydrogel with 2 mL bacterial suspension (10^8^ CFU/mL) was co-cultured at 37°C for 6 h. The bacterial suspension was collected, centrifuged at 1200 rpm for 10 min and washed twice with PBS (pH 7.4). The suspended liquid droplets of bacteria were placed on the silicon wafer and dried naturally until a white film visible to the naked eye is observed. After fixing the bacteria overnight with glutaraldehyde solution (2.5% PBS) at 4°C, rinse with PBS three times. The samples were then dehydrated with ethanol in gradient alcohol concentrations (25%, 50%, 75%, 90% and 100%). The morphology of bacteria was observed by SEM after gold spraying.

### Antioxidant property

Take 0.2 g of SGH, Ca@Q-E@SGH and a concentration of 200 μL of Ca@Q-E (100 μg/mL) and add them to 1.8 mL of DPPH and ABTS free radicals, respectively. Add 200 μL of Vc as the positive control group. The absorption spectra were recorded at 517 nm and 750 nm using a Shimadzu UV-3600 plus spectrophotometer (Japan), followed by calculation of DPPH and ABTS radical scavenging activities according to the formula (6):


(6) $$\text{DPPH/ABTS scavening activity (\%)}=(\frac{A_{S}-A_{N}}{A_{P}-A_{N}})\times 100\%$$

In this equation, As represents the sample’s absorbance, while *A*_N_ and *A*_P_ correspond to the negative control and positive control absorbance measurements, all acquired at either 517 nm or 750 nm wavelength.

### Biocompatibility

To assess hydrogel biocompatibility, human umbilical vein endothelial cells (HUVECs) were cultured on sterilized hydrogel surfaces with an initial seeding density of 1 × 10^4^ cells per well. Subsequently, the cells are cultured for 24 h and 48 h. During this period, their proliferation and viability on the hydrogels were assessed by live/dead staining and CCK-8 assay.

Cell cytoskeleton: Following a 72-hour co-culture period of HUVECs with different hydrogel constituents, cellular visualization was performed through dual fluorescence staining using rhodamine-phalloidin for F-actin cytoskeletal labeling and DAPI (4',6-diamidino-2-phenylindole, Life Technologies) for nuclear counterstaining.

### Ca@Q-E@SGH hydrogel regulates the microenvironment *in vitro*

HUVECs were seeded into 12-well plates (1 × 10^4^ cells per well) and cultured overnight. H_2_O_2_ (400 μM) and different component materials were added for incubation for 2 h. To assess cellular responses under oxidative stress conditions, HUVECs were first labeled with 10 μM DCFH-DA and HIF-1α antibodies for 30 min, followed by fluorescence imaging using a Leica DMi8-s inverted microscope. After 24 h of culture on hydrogel substrates, cell proliferation and viability were assessed by combining live/dead staining with a CCK-8 assay.

To establish M1-polarized macrophages for assessing hydrogel immunomodulation, RAW264.7 cells were first stimulated with lipopolysaccharide (LPS) for 48 h. Following this induction period, test hydrogels were incorporated into the culture system for another 48 h of incubation. Immunophenotyping was performed by first treating cells overnight at 4°C with primary antibodies specific for M1 (CD86) or M2 (CD206) surface markers, then exposing them to fluorescent secondary antibodies for 30 min. Nuclei were subsequently stained with DAPI prior to imaging with an inverted fluorescence microscope to characterize macrophage subpopulations. Parallel flow cytometry experiments were conducted to quantitatively assess macrophage polarization. Following the same LPS and material treatment protocol, cells were harvested and triple-stained with F4/80-FITC (pan-macrophage marker), CD86-PE (M1 subset), and CD206-APC (M2 subset) antibodies prior to flow cytometric analysis. The levels of both pro-inflammatory (IL-1β, TNF-α) and anti-inflammatory (IL-10, Arg-1) mediators in the culture supernatants were quantified by ELISA.

### Tube formation assay

The angiogenic potential of hydrogels was assessed through an *in vitro* Matrigel-based tube formation assay. Briefly, 96-well plates were pre-coated with Matrigel matrix and incubated at 37°C for 30 min to facilitate polymerization. Following matrix solidification, HUVECs were seeded and maintained for 10 h in serum-free medium supplemented with PBS (control), GH, SGH, or Ca@Q-E@SGH hydrogel extracts, after which tubular structure development was microscopically evaluated.

### *In vivo* study

The experimental work with animals was performed following the ethical guidelines established by the National Research Council for laboratory animal use and was officially sanctioned by the Animal Ethics Committee of Guangzhou Dermatology Hospital (No. N2025-40003). Sprague-Dawley (SD) rats with a body weight of approximately 220–250 g were used. Firstly, after being fed a high-fat and high-sugar diet for 2 weeks, a diabetic model was established by injecting streptozotocin (55 mg/kg, product of Thermo Fisher Scientific). Prior to modeling, the dorsal surgical area of all anesthetized rats was shaved. To simulate the clinically susceptible-to-infection state of diabetic wounds, the instruments were cleaned and disinfected, but extreme aseptic techniques were not employed, in order to better recapitulate the delayed healing characteristic of human diabetic wounds. Circular wounds with a diameter of 15 mm were made using tools, and the wounds were treated with PBS, GH, SGH and Ca@Q-E@SGH hydrogels. The progression of wound healing was monitored at specified intervals (days 0, 3, 5, 7, 9 and 14 post-operation), with wound area quantification performed using ImageJ analysis software. On day 14, euthanized animals underwent tissue harvest from the wound site, where excised skin specimens were divided for processing. A portion of samples were preserved in 4% paraformaldehyde solution for comprehensive histological evaluation, including: structural analysis through H&E and Masson’s trichrome staining and molecular characterization via immunofluorescence detection of MMP-9, TNF-α, IL-10, IL-6, α-SMA and CD31 markers.

### Statistical analysis

Statistical analysis was performed using SPSS 27 (SPSS Inc., Chicago, IL, USA), with all experimental data representing at least three independent replicates (*n *≥ 3). Quantitative results are expressed as mean ± standard deviation, with normalized data specifically indicated in corresponding figure legends. Multiple group comparisons were conducted by one-way ANOVA followed by Tukey’s *post hoc* test, with significance levels denoted as *P < 0.05, **P < 0.01 and ***P < 0.001 for statistically significant differences.

## Results and discussion

### Preparation and characterization of borate ester bond responsive self-oxygen-providing Ca@Q-E nanoparticle platform

**Figure 2:**
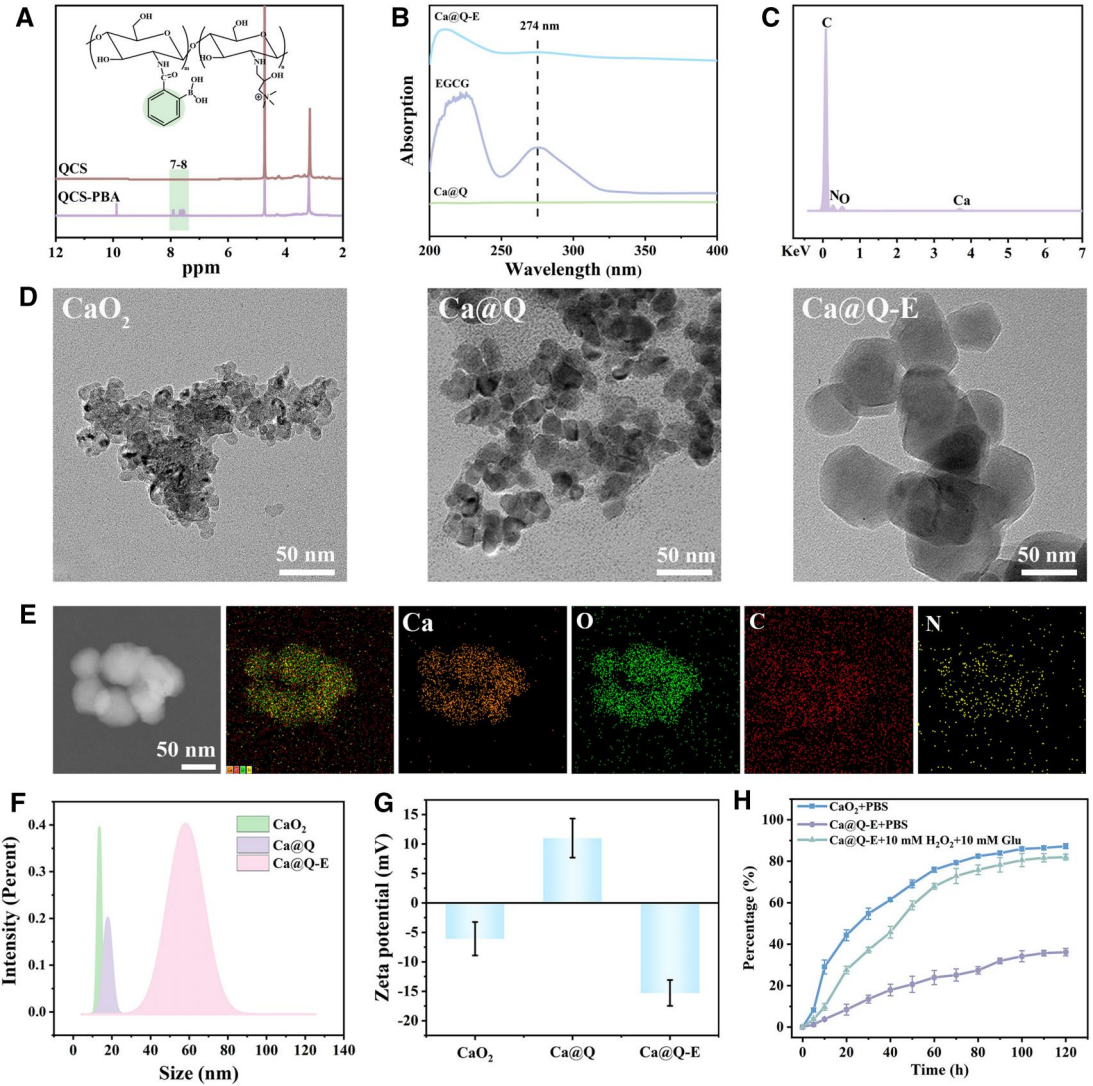
(**A**) The ^1^H NMR spectrums of QCS and QCS-PBA. (**B**) Ultraviolet-visible absorption spectra of Ca@Q, EGCG and Ca@Q-E. (**C**) EDS spectra of Ca@Q-E. (**D**) TEM images of CaO_2_, Ca@Q and Ca@Q-E. (**E**) The distribution of elements in Ca@Q-E. (Ca, O, C, N). (**F**) Particle size distribution map and (**G**) zeta potential of CaO_2_, Ca@Q and Ca@Q-E. (**H**) Oxygen release ratio of Ca@Q and Ca@Q-E.

The CaO_2_ nanoparticles, serving as the self-oxygenating core, were synthesized via the Calcium Hydroxide-Hydrogen Peroxide Method [24]. A composite oxygen-releasing system (Ca@Q) was constructed by encapsulating CaO_2_ with PBA-modified QCS (QCS-PBA). Subsequently, the polyphenolic structure of EGCG was utilized to form ROS/glucose-responsive dynamic borate ester bonds with phenylboronic acid. The concentration of Ca^2+^ in the Ca@Q-E nanoparticles was determined to be 161.74 μg/mg by ICP-OES. The successful synthesis of QCS-PBA was confirmed by ^1^H NMR and FT-IR spectroscopy. As evidenced by the ^1^H NMR spectrum (Figure 2A), the characteristic signals at 7–8 ppm were attributed to the aromatic protons of PBA. The FT-IR spectrum (Supplementary Figure S1) further supported this conclusion, showing the N-H bond at 3454 cm^−1^ shifts to 3417 cm^−1^, indicating that more primary amines have been replaced to form secondary amines and the appearance of a new peak at 1334 cm^−1^, which is characteristic of the B-O bond. This indicates that QCS-PBA was successfully prepared through the amide reaction, and the substitution degree of PBA was calculated to be 38.84%. UV-Vis spectroscopy (Figure 2B) revealed a characteristic absorption peak at 274 nm for EGCG in the Ca@Q-E nanoparticles. TEM analysis (Figure 2D) demonstrated that pristine CaO_2_ exhibited aggregated spherical morphology (average diameter: 13.50 nm), while QCS-PBA encapsulation (Ca@Q) significantly improved particle dispersion (average diameter: 17.80 nm). After EGCG loading (Ca@Q-E), the particle size increased to 40.08 nm (Figure 2F). Elemental mapping confirmed predominant Ca and O distribution in the nanoparticle core, while surface C and N signals verified successful QCS-PBA and EGCG modification (Figure 2C and E). Zeta potential evolution (Figure 2G and Supplementary Table S1) further evidenced the stepwise assembly: bare CaO_2_ showed negative potential (−15.2 mV), QCS-PBA coating (Ca@Q) induced positive shift (+8.5 mV), and subsequent EGCG loading (Ca@Q-E) restored negative potential (−12.7 mV), confirming successful electrostatic interaction-driven layer-by-layer construction. Oxygen release profiling (Figure 2H and Supplementary Figure S2) revealed that bare CaO_2_ exhibited a rapid oxygen release, whereas Ca@Q-E showed significantly sustained-release kinetics, with a cumulative release of 36.17 ± 1.79% over 120 h. To verify the ROS/glucose-responsive release of Ca@Q-E in a simulated diabetic wound microenvironment, tests were conducted in PBS containing 10 mM H_2_O_2_ and 10 mM glucose. Under these conditions, the cumulative oxygen release reached 81.99 ± 1.40%. These results confirm that the QCS-PBA coating effectively modulates the decomposition kinetics of CaO_2_ to achieve sustained oxygen delivery, and the ROS/glucose-responsive boronate ester bonds contribute to the intelligent regulation of oxygen release.

### Preparation and characterization of ROS/glucose-responsive anti-pollution Ca@Q-E@SGH hydrogel

**Figure 3:**
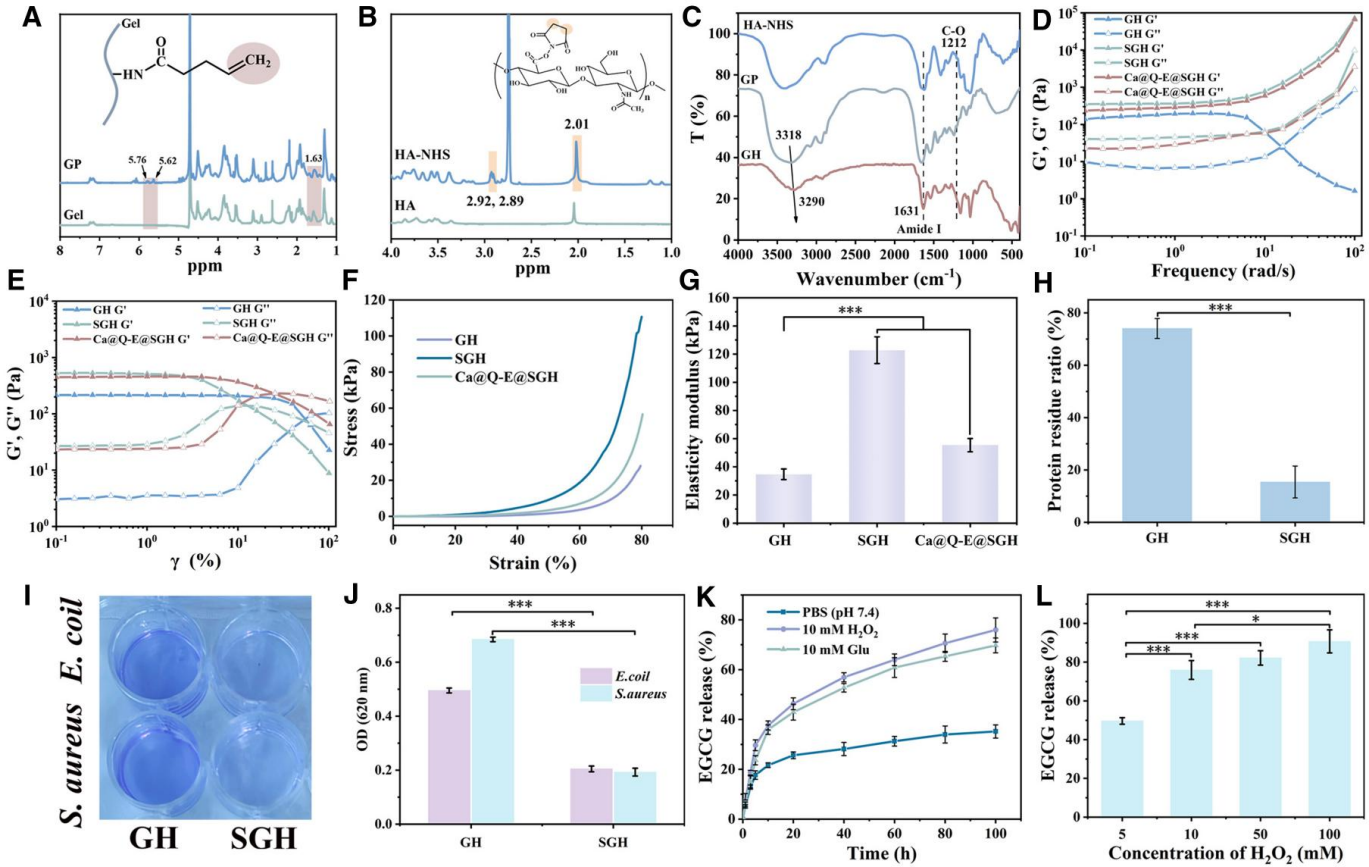
(**A**) The ^1^H NMR spectrums of Gel and GP. (**B**) The ^1^H NMR spectrums of HA and HA-NHS. (**C**) FT-IR spectrums of HA-NHS, GP and GH. (**D**) Strain scanning curve of GH, SGH and Ca@Q-E@SGH. (**E**) Frequency scanning curve of GH, SGH and Ca@Q-E@SGH. (**F**) Compressive stress-strain curves and (**G**) compressive elasticity modulus of GH, SGH and Ca@Q-E@SGH. (**H**) The residual ratios of bovine serum proteins on GH and SGH were determined by the BCA method. (**I**) OD values and (**J**) crystal violet-stained images of residual *E. coli* and *S. aureus* on GH and SGH. (**K**) The release curves of EGCG released by Ca@Q-E@SGH in PBS, H_2_O_2_ and Glu media, respectively. (**L**) Release ratio of EGCG from Ca@Q-E@SGH in media with different concentrations of H_2_O_2_.

The Ca@Q-E@SGH bilayer hydrogel features a lower layer composed of Gel-PA and HA-NHS (GH hydrogel) loaded with Ca@Q-E nanoparticles. The GH network forms through amide bond cross-linking between amino groups of Gel-PA and NHS ester groups of HA-NHS. The upper anti-fouling layer is constructed via UV-initiated polymerization of carbon-carbon double bonds in GH with sulfobetaine methacrylate (SBMA). The successful grafting of the double bond onto Gel-PA was confirmed by ^1^H NMR and FT-IR spectroscopy. The ^1^H NMR spectrum (Figure 3A) showed characteristic peaks at 5.62 and 5.76 ppm, which are assigned to the vinyl protons of PA, and it was calculated that the substitution degree of PA was 12.28%. The FT-IR spectrum further confirmed that PA was grafted onto Gel through amide reaction (Supplementary Figure S3), revealing a shift of the N-H stretching vibration from 3422 cm^−1^ to 3318 cm^−1^, along with a significant intensification of the amide I and amide II bands at 1654 cm^−1^ and 1545 cm^−1^, respectively. HA-NHS showed characteristic NHS five-membered ring proton peaks at 2.89 and 2.92 ppm (Figure 3B). The substitution degree of NHS in HA was calculated to be 30.75%. Subsequently, the cross-linking mechanism of the GH hydrogel was confirmed by FT-IR spectroscopy. As shown in Figure 3C, the shift of the N-H stretching vibration from 3318 cm^−1^ to 3290 cm^−1^, coupled with the appearance of the amide I band at 1631 cm^−1^ and the disappearance of the C-O characteristic peak of NHS at 1212 cm^−1^, collectively verified that the cross-linking occurred via an amidation reaction between HA-NHS and the amino groups on GP. Subsequently, the formed GH hydrogel was further modified via UV-induced cross-linking to fabricate SGH hydrogel with an SBMA anti-fouling coating, ultimately yielding the Ca@Q-E@SGH hydrogel. The colloidal stability of Ca@Q-E nanoparticles was further investigated to evaluate their suitability for biomedical applications. As depicted in Supplementary Figure S4, upon incubation with H_2_O_2_, the nanoparticles underwent rapid degradation by day 7, resulting in a clear solution without any observable aggregation. This indicates their desirable responsiveness to an oxidative microenvironment. Moreover, SEM-EDS mapping analysis of the Ca@Q-E@SGH hydrogel was conducted to examine the distribution of the nanoparticles within the scaffold. The homogeneous distribution of the Ca element (Supplementary Figure S5) confirms the excellent dispersibility of Ca@Q-E nanoparticles throughout the hydrogel matrix. These results collectively demonstrate the outstanding colloidal stability of the nanoparticles, providing a solid foundation for their use in subsequent *in vivo* studies. Rheological characterization was performed to evaluate the mechanical properties of the hydrogels, which are critical for wound dressing applications. The frequency sweep (Figure 3D) revealed that the SGH hydrogel possessed the highest storage modulus (G'), indicating superior mechanical strength. However, its relatively low fracture point in the strain sweep (Figure 3E) suggested increased brittleness after UV cross-linking. In contrast, the Ca@Q-E@SGH hydrogel exhibited a moderate modulus, coupled with a significantly higher strain tolerance. This combination of adequate strength and excellent deformability is ideal for wound management.

Compression tests demonstrated the hydrogel’s flexibility for wound conformability (Figure 3F). At 80% strain, neither GH, SGH nor Ca@Q-E@SGH fractured. Notably, SGH (with SBMA coating) exhibited optimal mechanical properties (110 kPa stress at 80% strain, compressive modulus 122.78 ± 9.47 kPa) due to its dual cross-linking network combining amide bonds and high-energy C-C covalent bonds (from double bond polymerization). In contrast, GH with single amide cross-linking showed lower modulus (34.66 ± 3.74 kPa). The incorporation of Ca@Q-E nanoparticles reduced Ca@Q-E@SGH’s modulus to 55.39 ± 4.70 kPa (Figure 3G) due to steric hindrance effects decreasing cross-linking density. Furthermore, Ca@Q-E@SGH demonstrated excellent elastic recovery performance during 30 compression cycles (Supplementary Figure S6). The stress–strain curve was highly repetitive and the stress was stable. This outstanding elastic recovery ability enables it to adapt to the mechanical changes in the dynamic environment of the wound, laying an important mechanical foundation for its subsequent biomedical applications *in vivo*.

Hydrogel materials designed for wound repair demonstrate significant anti-fouling properties that effectively prevent bacterial adhesion and contamination, thereby providing an excellent physical barrier for wound protection. In this study, the anti-fouling capability of the Ca@Q-E@SGH hydrogel primarily originates from the zwitterionic structure of its SBMA component, which functions through the formation of a dense hydration layer and charge neutralization effects. Protein adsorption assays (BCA method) revealed (Figure 3H) that, based on the standard curve (y = 0.11608x + 0.2562, R^2^ = 0.99907, Supplementary Figure S7), the residual protein adsorption rate on the SGH surface (15.39 ± 0.06%) was significantly lower than that of the control GH hydrogel (74.02 ± 0.03%). Bacterial adhesion tests (crystal violet staining) were conducted using *E. coli* and *S. aureus* for evaluation. As shown in Figure 3I measurements at 620 nm via a microplate reader confirmed that bacterial retention on the GH surface was substantially higher than that on SGH. This difference was also visually evident in the macroscopic images (Figure 3J), where minimal crystal violet staining remained on the SGH surface, further validating its superior anti-fouling performance.

The smart controlled-release system developed in this study enables dynamic drug release in response to the wound microenvironment. Specifically, the release behavior of EGCG from the Ca@Q-E@SGH hydrogel is regulated by the ROS/glucose dual-responsive characteristics of its borate ester bonds. As shown in Figure 3K, the cumulative release of EGCG was significantly higher in media containing 10 mM H_2_O_2_ and 10 mM glucose (Glu) compared to PBS (pH 7.4), reaching (75.94 ± 4.84%) and (69.74 ± 2.96%), respectively after 100 h of incubation. Furthermore, EGCG release exhibited concentration-dependent responsiveness to H_2_O_2_ (Figure 3L). In 100 mM H_2_O_2_ medium, the cumulative release reached (90.72 ± 5.97%) after 100 h. This responsive release mechanism originates from oxidative cleavage of borate ester bonds induced by H_2_O_2_ attack, combined with competitive binding between glucose and boronic acid groups. Notably, this process not only releases EGCG but also helps reduce local free glucose concentration. In conclusion, the Ca@Q-E@SGH hydrogel demonstrates both excellent deformability and outstanding anti-fouling properties, providing an effective physical barrier for wound protection. Its ROS/glucose-responsive EGCG release function further synergistically accelerates the healing process of diabetic wounds.

### The antibacterial and antioxidant properties of Ca@Q-E@SGH hydrogel

**Figure 4:**
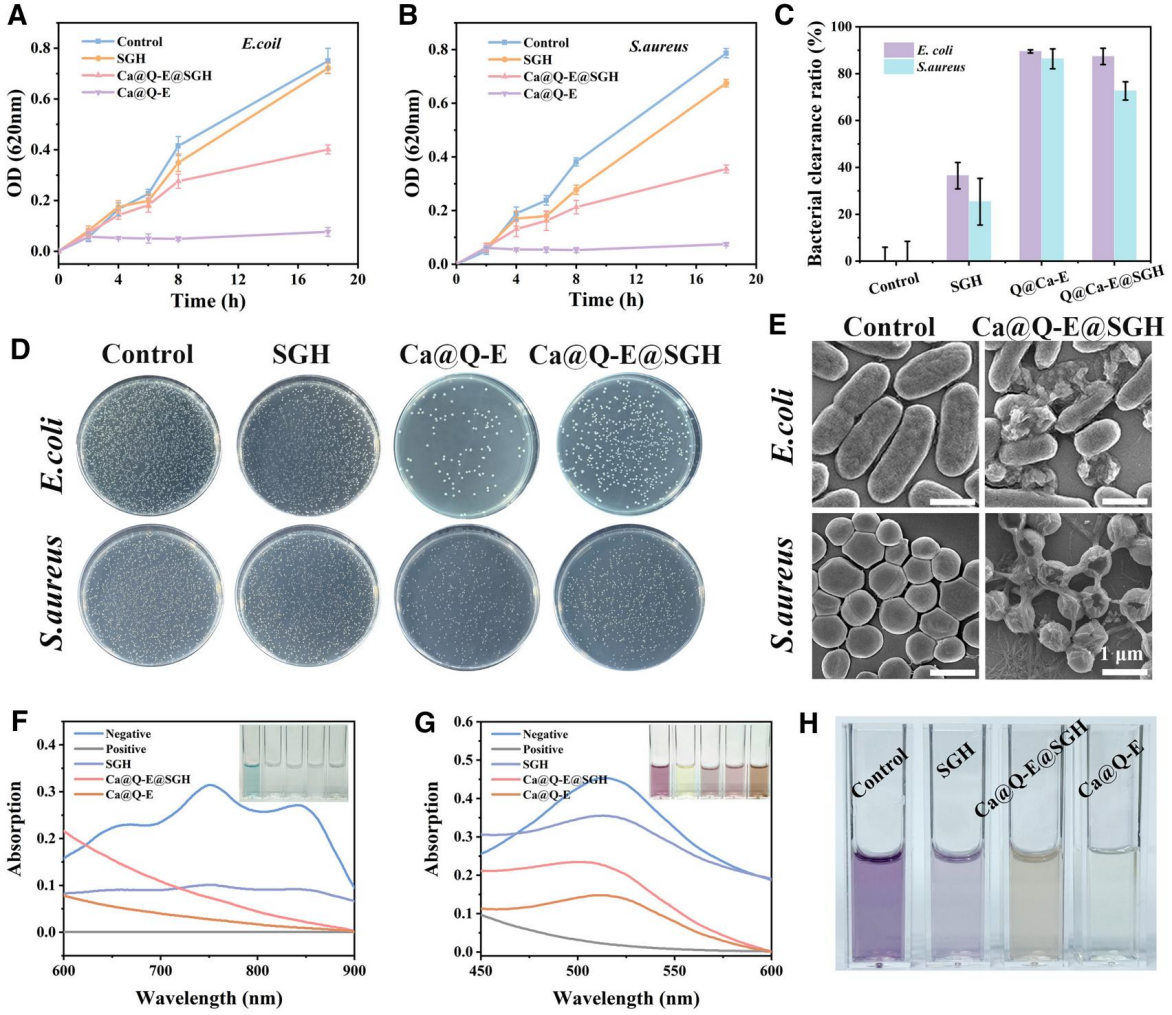
Growth curves of (**A**) *E. coli* and (**B**) *S. aureus* post-incubation with various materials. (**C**) Bacterial clearance ratios for *E. coli* and *S. aureus* following 8-h incubation with different materials. (**D**) Representative colony images of *E. coli* and *S. aureus* after 8-h incubation with various materials. (**E**) SEM micrographs of *E. coli* and *S. aureus* before and after treatment. (**F**) UV-Vis spectra of ABTS treated with different components. (**G**) UV-Vis spectra of DPPH treated with different components. (**H**) Physical image of the salicylic acid color reaction.

In the field of wound repair, endowing biomaterials with antibacterial properties is crucial for preventing infection and promoting tissue regeneration [33]. This study systematically evaluated the antibacterial activity of Ca@Q-E@SGH hydrogel. Dynamic monitoring of bacterial growth curves (Figure 4A and B) after co-incubation of PBS, GH, SGH and Ca@Q-E@SGH with *E. coli* and *S. aureus* revealed that the Ca@Q-E nanoparticle group rapidly inhibited bacterial proliferation, while Ca@Q-E@SGH gradually demonstrated significant antibacterial effects after 8 h of incubation due to the sustained-release characteristics of active components. Further quantification of antibacterial efficacy was performed using plate counting method (Figure 4D), with clearance rates calculated based on colony counts (Figure 4C). Quantitative analysis showed that the Ca@Q-E group achieved high clearance ratios of (89.48 ± 0.68%) for *E. coli* and (86.32 ± 4.26%) for *S. aureus*. In contrast, Ca@Q-E@SGH exhibited slightly reduced clearance ratios (*E. coli*: 87.37 ± 3.46%; *S. aureus*: 72.65 ± 3.89%) due to the physical encapsulation of Ca@Q-E nanoparticles delaying active component release. To observe the morphological and structural changes in bacteria for elucidating the antibacterial mechanism of the Ca@Q-E@SGH, SEM was employed to examine the samples from both the control group and the group treated with the Ca@Q-E@SGH. As illustrated in Figure 4E, the treated group of *E. coli* and *S.aureus* exhibits ruptured cell membranes and leakage of cellular contents, a stark contrast to the Control group where the bacteria maintain a smooth surface and plump morphology. To elucidate the source of the material’s antibacterial activity, we conducted agar plate assays with EGCG, CaO_2_, and the Ca@Q-E composite individually (Supplementary Figure S8). The results clearly demonstrated that CaO_2_ alone exhibited only marginal inhibitory effects, whereas EGCG served as the primary antibacterial agent. Notably, the Ca@Q-E composite showed the strongest antibacterial activity, indicating a synergistic effect between CaO_2_ and EGCG. Furthermore, the antibacterial efficacy of Ca@Q-E nanoparticles was found to be concentration-dependent, with higher concentrations leading to progressively greater suppression of bacterial growth (Supplementary Figure S9). Collectively, these findings confirm that the antibacterial property of the Ca@Q-E@SGH hydrogel originates primarily from the incorporated Ca@Q-E nanoparticles. The key component, EGCG, plays a central role by damaging bacterial membrane integrity and interfering with metabolic pathways. This action is synergistically enhanced by Ca^2+^, which contributes by altering the microenvironment (e.g., inducing hypoxia and high osmolarity) and directly disrupting membrane structures [34, 35].

In diabetic wound repair, the antioxidant design of biomaterials holds critical therapeutic value by targeting the characteristic oxidative stress microenvironment [36]. The developed Ca@Q-E@SGH hydrogel exerts potent antioxidant effects through the catechol groups in EGCG, operating via multiple mechanisms including electron transfer and free radical neutralization. The 2,2'-azino-bis 3-ethylbenzothiazoline-6-sulfonic acid (ABTS^+^•) radical scavenging assay (Figure 4F) demonstrated characteristic absorption peaks at 750 nm with blue–green coloration. Upon reaction with various antioxidants (Vc, SGH, Ca@Q-E@SGH and Ca@Q-E), the solution decolorized significantly as radicals were reduced to colorless ABTS, accompanied by peak disappearance. Quantitative analysis (Supplementary Figure S10) revealed ABTS^+^• scavenging rates of (76.84 ± 3.44%) for Ca@Q-E@SGH and (91.05 ± 0.44%) for Ca@Q-E, confirming EGCG integration substantially enhanced electron-donating capacity. In the 1,1-diphenyl-2-picrylhydrazyl (DPPH•) assay (Figure 4G), the characteristic 560 nm absorption of the deep purple solution shifted to light yellow upon hydrogen atom transfer. Ca@Q-E exhibited (72.31 ± 1.38%) DPPH• scavenging (Supplementary Figure S11), demonstrating significant hydrogen-donating ability [37]. Additional hydrogen peroxide (H_2_O_2_) scavenging evaluation using the Fe^3+^-salicylate system (Figure 4H) showed both Ca@Q-E@SGH and Ca@Q-E effectively decomposed H_2_O_2_, maintaining light yellow coloration by inhibiting purple complex formation—directly addressing the clinical need for ROS elimination in diabetic wounds.

### Biological safety, oxidative stress regulation and vasculogenic-promoting functions of Ca@Q-E@SGH hydrogel

**Figure 5:**
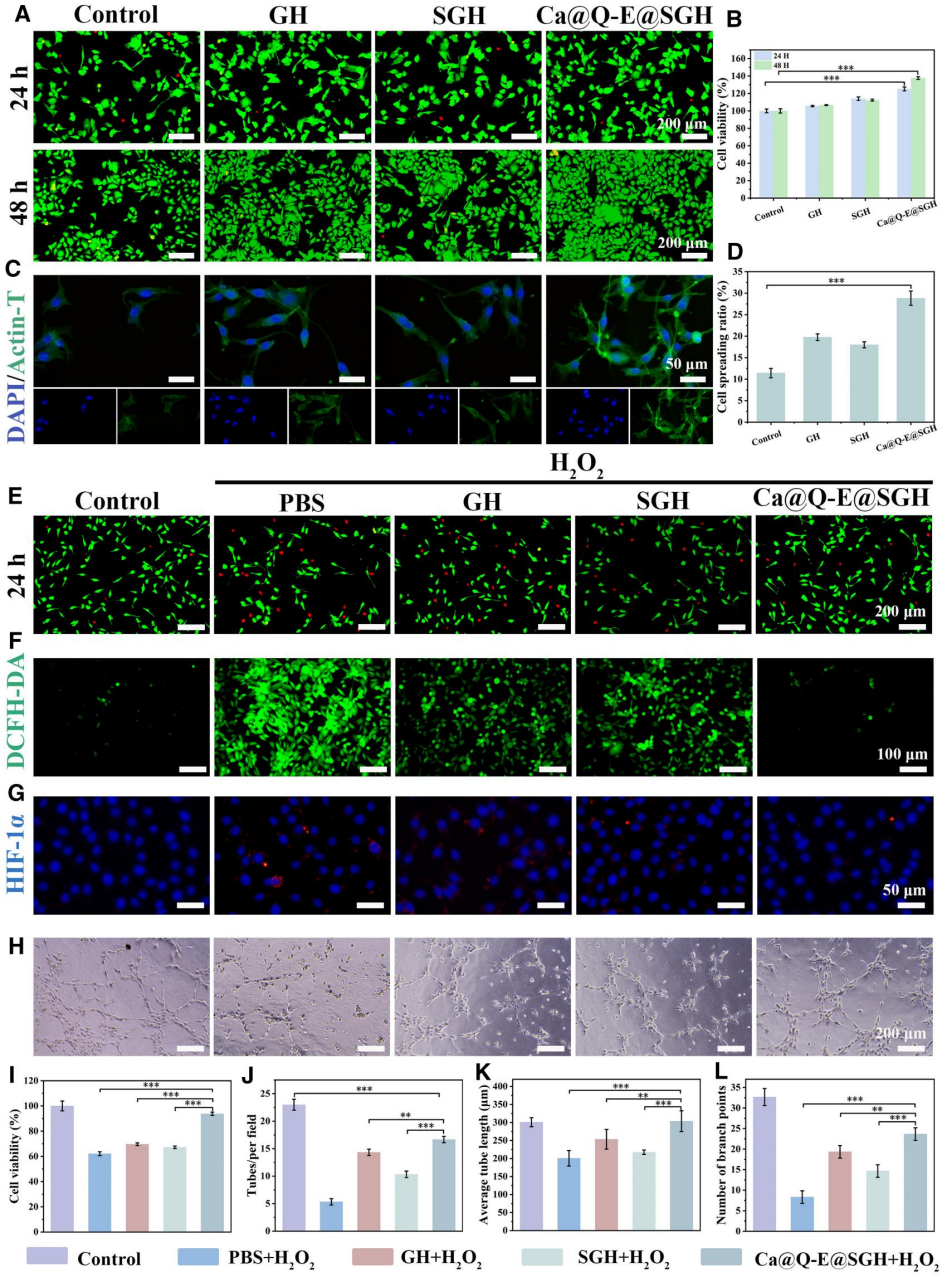
(**A**) Live and dead cell staining of HUVEC cells after co-culture with PBS, GH, SGH and Ca@Q-E@SGH for 24 h and 48 h, and (**B**) Cell viability determined by CCK-8 assay. (**C**) Cytoskeleton staining of HUVECs. (**D**) Quantification of cell spreading area. (**E**) Live and dead staining of HUVECs cultured in H_2_O_2_ medium with indicated treatments for 24 h. (**F**) DCFH-DA is used to measure the intracellular ROS levels. (**G**) HIF-1α fluorescence staining. (**H**) Representative image of HUVEC cells forming tubes. (**I**) CCK8 assay of HUVEC cells after co-culture with PBS, GH, SGH and Ca@Q-E@SGHG in H_2_O_2_ medium for 24 h. Angiogenic capacity on Matrigel: (**J**) Number of tube formation. (**K**) Average tube length. (**L**) Number of branch points.

Biocompatibility serves as a pivotal determinant for clinical translation potential, directly influencing tissue regeneration efficiency and therapeutic safety [38]. The biosafety assessment employing live/dead staining and CCK-8 assays revealed excellent cellular compatibility (Figure 5A). HUVECs cultured with GH, SGH and Ca@Q-E@SGH exhibited significantly increased cell density with minimal dead cell population, where Ca@Q-E@SGH demonstrated remarkable pro-proliferative capacity achieving 137.62 ± 1.52% cell viability at 48 h (***P < 0.001, Figure 5B). Cytoskeletal staining further elucidated material-mediated cellular responses (Figure 5C). Cells on Ca@Q-E@SGH displayed multipolar extension morphology with homogeneous F-actin stress fiber distribution. Quantitative analysis confirmed enhanced cell spreading ratio (Figure 5D), indicating the material surface favorably supports cellular adhesion and functional maintenance.

To simulate the oxidative stress microenvironment of diabetic wounds, an HUVECs oxidative damage model was established using H_2_O_2_ stimulation. Live/dead staining results (Figure 5E) demonstrated that after 24 h treatment with Ca@Q-E@SGH, cell density significantly increased compared to the Model group, with substantially reduced dead cell proportion. Quantitative viability analysis (Figure 5I) confirmed recovery to near-normal levels compared with Control group, outperforming both GH and SGH groups (**P < 0.01). DCFH-DA fluorescent probe detection (Figure 5F) provided mechanistic insight: while the Model group exhibited intense green fluorescence (indicating ROS burst), Ca@Q-E@SGH treatment reduced fluorescence intensity by (72.3 ± 5.1%) versus Model (***P < 0.001, Supplementary Figure S12), with visual assessment showing comparable levels to the Control group. These findings conclusively validate the system’s exceptional ROS-scavenging capacity.

Hypoxia-inducible factor-1α (HIF-1α), a core transcription factor for cellular adaptation to hypoxic environments [39]. In the Model group, significantly enhanced HIF-1α fluorescence signals with dense punctate distribution were observed, whereas the Ca@Q-E@SGH group demonstrated substantial reduction in fluorescence intensity (***P < 0.001, Supplementary Figure S13), indicating that the self-oxygenating system in Ca@Q-E@SGH hydrogel effectively regulates HIF-1α homeostasis (Figure 5G). Early upregulation of HIF-1α facilitates angiogenesis; however, the inflammatory state induced by sustained hypoxic stress leads to the formation of dysfunctional and leaky vasculature. A normalized microenvironment is more conducive to initiating proper angiogenesis [40]. As shown in Figure 5H, where HUVECs in the Model group aggregated into clusters forming only fragmented short tubes, while the Ca@Q-E@SGH group developed interconnected complex tubular networks resembling the Control group morphology. Although the tube formation number and branch points in Ca@Q-E@SGH group (Figure 5L) remained statistically different from the Control, they significantly exceeded those in GH (**P < 0.01) and SGH (***P < 0.001) groups. Notably, the average tube length in Ca@Q-E@SGH group (303 ± 29 μm) showed no significant difference from the Control group (301 ± 12 μm) (Figure 5K). These experimental results collectively demonstrate that Ca@Q-E@SGH possesses excellent biocompatibility, potent ROS-scavenging capacity, and hypoxia microenvironment modulation capability, which significantly promotes HUVECs vascular network formation, thereby establishing a solid foundation for subsequent *in vivo* studies on diabetic wound healing.

### Immune regulation of the microenvironment of Ca@Q-E@SGH hydrogel

**Figure 6:**
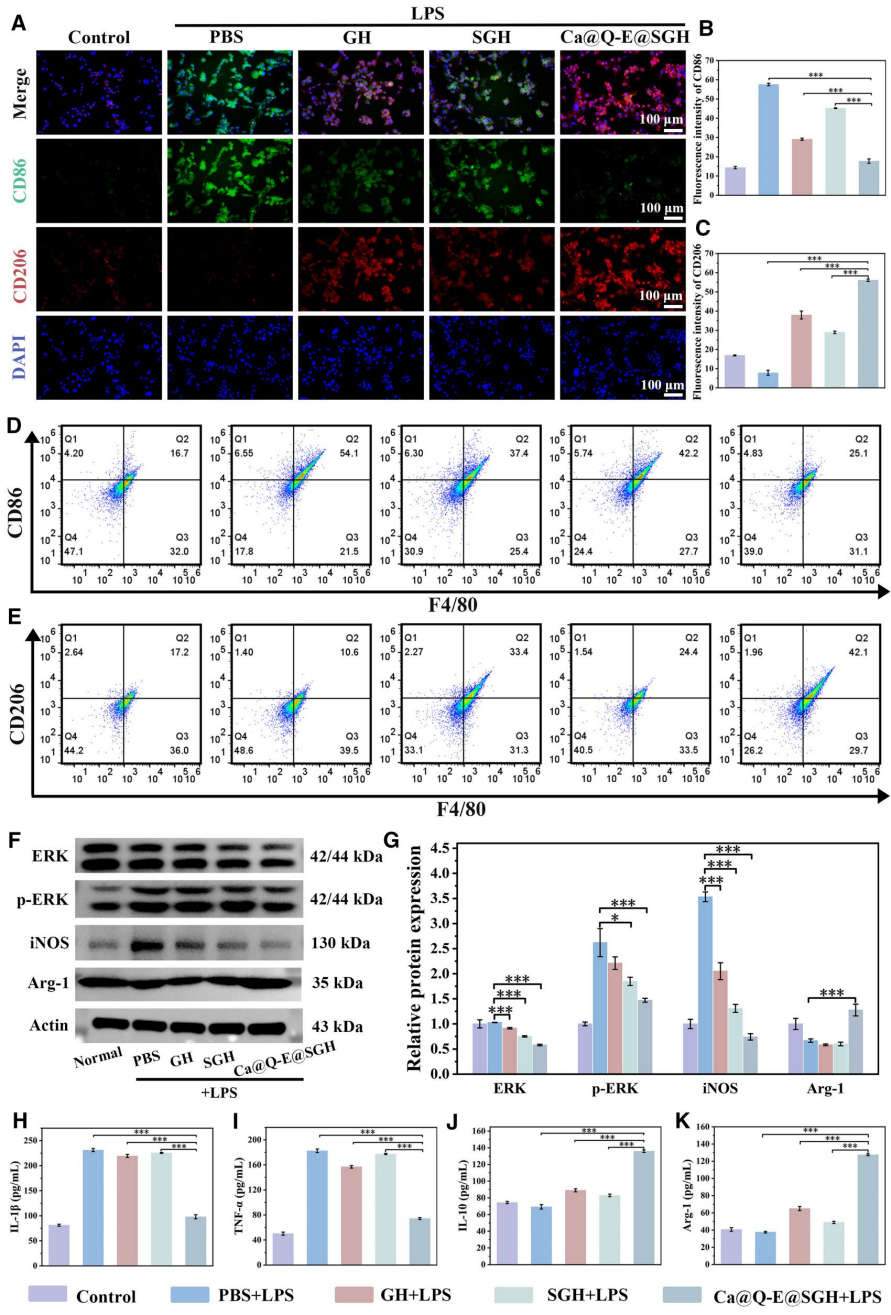
(**A**) Immunofluorescence staining of CD86 and CD206 in RAW264.7 cells after co-incubation with PBS, GH, SGH and Ca@Q-E@SGH in the presence of LPS, fluorescence intensity statistics of (**B**) CD86 and (**C**) CD206 and (**D–E**) flow cytometry analysis. (**F**) WB detection of the expression of ERK, p-ERK, iNOS and arg-1. (**G**) Quantitative statistics of relative protein expression. ELISA quantification of pro-inflammatory factors (**H**) IL-1β, (**I**) TNF-α and anti-inflammatory factors (**J**) IL-10, (**K**) Arg-1 in RAW264.7 cells.

Macrophage phenotypic polarization, particularly the transition from pro-inflammatory M1 to reparative M2 phenotype, serves as a pivotal regulator of inflammation resolution and tissue regeneration processes [41]. This critical biological mechanism has positioned targeted macrophage polarization as a highly promising therapeutic strategy for diabetic wound repair. In the present study, we established an M1-polarized macrophage model by stimulating RAW264.7 cells with lipopolysaccharide (LPS), with subsequent phenotypic characterization through immunofluorescence staining of M1 marker CD86 (green fluorescence) and M2 marker CD206 (red fluorescence). As demonstrated in Figure 6A, control cells exhibited minimal fluorescence signals for both markers, while the Model group showed robust CD86 expression confirming successful M1 polarization. Strikingly, Ca@Q-E@SGH hydrogel treatment induced pronounced CD206 positivity coupled with marked CD86 suppression, providing clear visual evidence of its M1-to-M2 polarization capability. Subsequently, statistical analysis of CD86 and CD206 fluorescence intensity (Figure 6B and C) revealed a consistent trend: the Ca@Q-E@SGH group exhibited a significantly higher CD206 fluorescence intensity compared to the Model group, while CD86 showed the opposite pattern. Flow cytometric analysis (Figure 6D and E) and statistical analysis of the M1/M2 phenotype ratio (Supplementary Figure S14) validated these observations, revealing that Ca@Q-E@SGH treatment significantly reduced M1-double-positive cell populations to near-control levels while maximizing M2-double-positive subsets.

To further investigate the mechanism by which Ca@Q-E@SGH promotes macrophage reprogramming toward the M2 phenotype, we conducted additional experiments. Western blot (WB) analysis revealed that Ca@Q-E@SGH significantly suppressed ERK phosphorylation (Figure 6F), downregulated the expression of the M1 marker iNOS, and upregulated the expression of the M2 marker Arg-1 (Figure 6G). These findings preliminarily suggest that Ca@Q-E@SGH inhibits M1 polarization and promotes M2 polarization of macrophages via suppression of the ERK signaling pathway. Given the close relationship between macrophage polarization states and their secretory profiles, we further quantified the expression levels of key inflammatory cytokines using ELISA. The results demonstrated that Ca@Q-E@SGH treatment significantly inhibited the release of pro-inflammatory cytokines (IL-1β and TNF-α; ***P < 0.001) while upregulating the expression of anti-inflammatory/reparative factors (IL-10 and Arg-1, ***P < 0.001 compared to the Model group) (Figure 6H–6K). Previous studies have reported that EGCG can mitigate diabetes-induced mitochondrial dysfunction by reducing ROS generation and inhibiting the NF-κB and ERK signaling pathways. In addition to directly modulating macrophage polarization through the restoration of mitochondrial autophagic flux, EGCG may also indirectly influence macrophage phenotype by restoring the immunomodulatory function of stem cells [42, 43]. In summary, this study systematically demonstrates that Ca@Q-E@SGH hydrogel effectively redirects macrophage polarization from the pro-inflammatory M1 phenotype toward the reparative M2 phenotype by inhibiting ERK phosphorylation. This phenotypic shift remodels the inflammatory cytokine milieu, thereby establishing an immunomodulatory microenvironment conducive to diabetic wound healing.

### The mechanism underlying the effects of the Ca@Q-E@SGH hydrogel in diabetic wound healing

**Figure 7:**
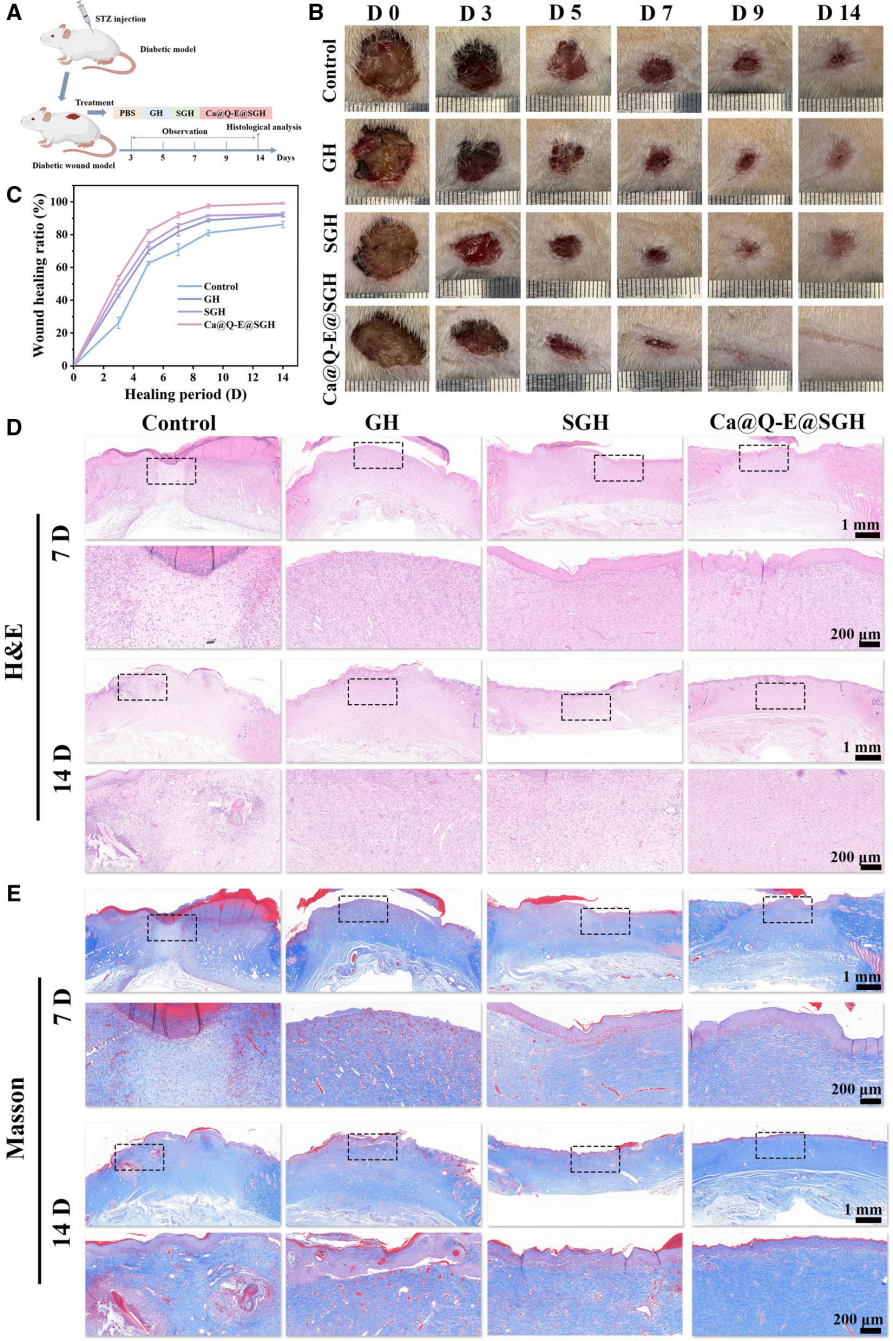
(**A**) Schematic illustration of hydrogel dressings treatment on bacterial-infected full-thickness wound defect model. (**B**) Photographs of the diabetic wound after treatment with PBS, GH, SGH and Ca@Q-E@SGH for different days. (**C**) Statistics of wound healing ratio. Pathological histological analysis (**D**) H&E staining and (**E**) Masson staining.

A type II diabetic rat model was established through streptozotocin (STZ) induction, followed by creation of full-thickness skin defects to systematically evaluate the therapeutic effects of different interventions on diabetic wound healing. Experimental groups received PBS (Control), GH, SGH, or Ca@Q-E@SGH treatments, with wound closure progression monitored dynamically on days 0, 3, 5, 7, 9 and 14 post-operation. Euthanasia and tissue collection were performed on days 7 and 14 for histopathological analysis (Figure 7A). Morphological documentation (Figure 7B) revealed that Ca@Q-E@SGH treatment initiated significant wound area reduction by day 5, achieving near-complete closure by day 9. Quantitative analysis (Figure 7C) confirmed superior healing efficacy, with Ca@Q-E@SGH reaching 99.10 ± 0.53% wound closure by day 14 versus 86.20 ± 2.00% in Controls (***P < 0.001). For a direct performance comparison, the wound healing ratios of our dual-responsive hydrogel were explicitly contrasted with recent state-of-the-art systems in Supplementary Table S2. The results demonstrate that our hydrogel achieves a wound closure rate comparable to, or even superior than, the most effective advanced dressings reported recently. H&E-stained sections (Figure 7D) demonstrated distinct healing patterns: day 7 Controls exhibited pathological hyperplasia with dense inflammatory infiltration and disorganized tissue architecture, while Ca@Q-E@SGH specimens showed mitigated inflammation despite mild hyperplasia. By day 14, although inflammation subsided across groups, Controls retained impaired regeneration markers (loose dermal structure, disordered fibroblasts), whereas Ca@Q-E@SGH group displayed near-physiological epidermal stratification with well-aligned fibroblasts and nascent microvessels in the dermis. At the same time, the number of hair follicle regenerations was also counted (Supplementary Figure S15). The Ca@Q-E@SGH group showed a significant hair follicle regeneration effect (***P < 0.001), confirming accelerated tissue regeneration.

Masson’s trichrome staining (Figure 7E) highlighted extracellular matrix (ECM) remodeling differences: while all groups showed increased collagen deposition by day 14, Controls presented fragmented, randomly oriented fibers. The SGH group, benefiting from anti-fouling barrier properties, surpassed GH in collagen density and organization. Notably, Ca@Q-E@SGH specimens exhibited optimal ECM reconstruction—dense, highly aligned collagen networks, collagen content accounted for 62.13 ± 1.88% (Supplementary Figure S16) with parallel bundle arrangements resembling native skin architecture. These findings demonstrate that Ca@Q-E@SGH hydrogel synergistically integrates antibacterial, anti-inflammatory, and pro-regenerative functions to achieve physiological ECM restoration.

### *In vivo* regulation of immune factors and angiogenesis control by Ca@Q-E@SGH hydrogel

**Figure 8:**
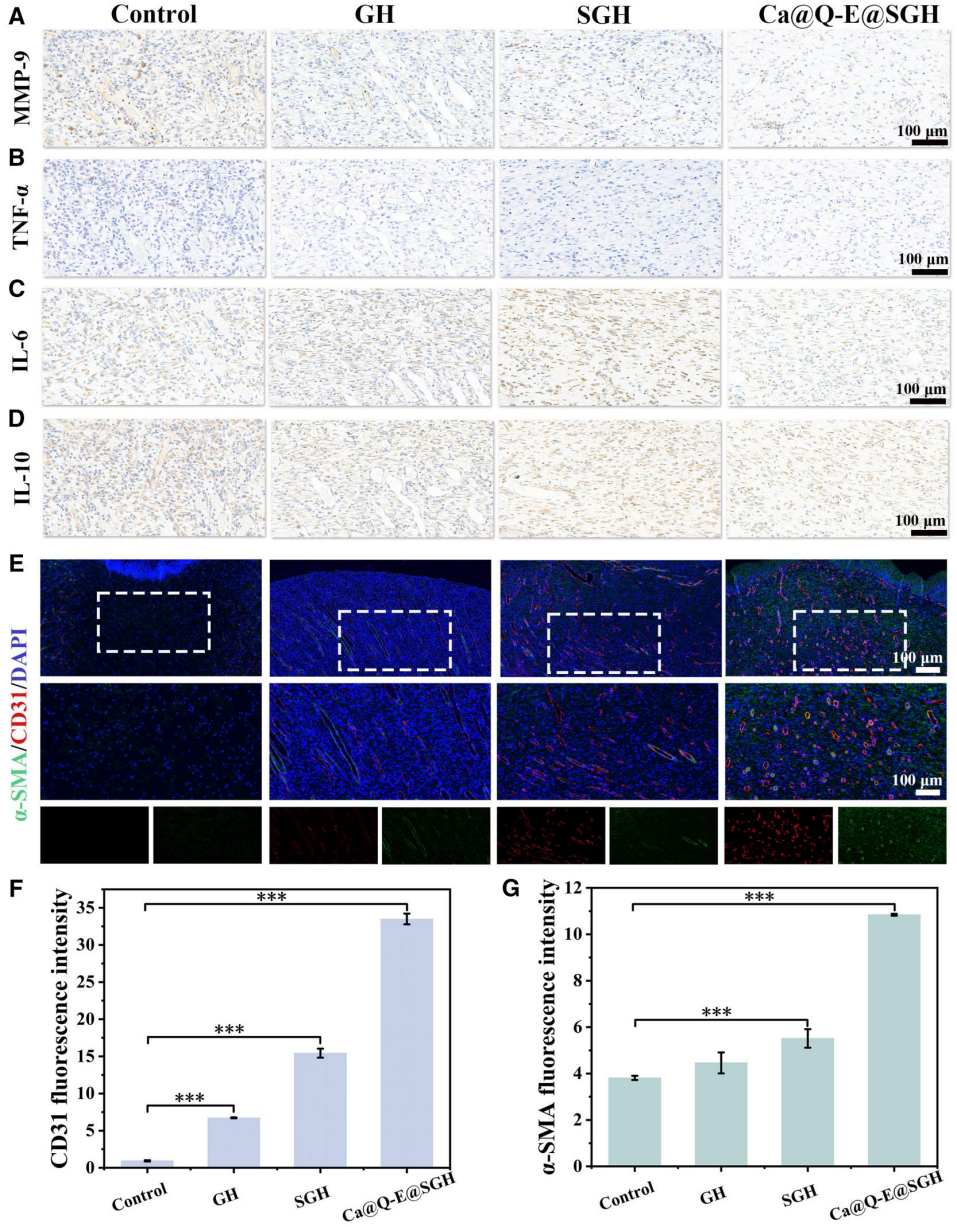
Immunohistochemical and immunofluorescence analyses on postoperative day 7 revealed the inflammatory response and vascular remodeling at the injury site. (**A**) MMP-9, (**B**) TNF-α, (**C**) IL-6, and (**D**) IL-10 expression assessed by immunohistochemistry. (**E**) Representative immunofluorescence images of α-SMA (smooth muscle cells) and CD31 (endothelial cells). Quantitative analysis of (**F**) CD31 and (**G**) α-SMA fluorescence intensity.

Immunohistochemical analysis of key molecular markers systematically revealed the wound microenvironment remodeling process mediated by Ca@Q-E@SGH treatment. Matrix metalloproteinase-9 (MMP-9), a pivotal mediator of extracellular matrix degradation, exhibited pathological over-activation in diabetic wounds due to chronic inflammation, leading to excessive breakdown of nascent basement membrane components. As shown in Figure 8A, strong MMP-9 immunopositivity was observed in Control wounds, whereas Ca@Q-E@SGH intervention significantly suppressed its expression—an effect mechanistically linked to the previously observed well-organized collagen deposition pattern, indicating enhanced tissue regeneration through matrix stabilization. The pro-inflammatory cytokines TNF-α and IL-6, serving as master regulators of inflammatory cascades, are overproduced by M1 macrophages and neutrophils, respectively. TNF-α compromises epithelial barrier integrity by inducing keratinocyte apoptosis, while IL-6 perpetuates inflammatory signaling networks that impede healing. Figure 8B and C demonstrated elevated TNF-α and IL-6 expression in Control tissues, correlating with histopathological observations of intensified inflammatory infiltration and delayed wound closure. In contrast, the significant downregulation of their coordination in the Ca@Q-E@SGH group explains molecularly the dual therapeutic effects of reducing inflammation and accelerating tissue regeneration (***P < 0.001, Supplementary Figure S17A–D). However, the anti-inflammatory cytokine IL-10, a signature secretion product of M2 macrophages, showed decreased levels following Ca@Q-E@SGH treatment (Figure 8D). This reduction may be attributed to the accelerated wound healing process promoted by Ca@Q-E@SGH, which facilitated the timely resolution of inflammation. This observation is consistent with the weak inflammatory signals observed in H&E staining, thereby diminishing the need for high levels of IL-10-mediated compensatory suppression.

Neovascularization recruits macrophages and other monocytes to wound sites while delivering essential oxygen, nutrients and growth factors to accelerate healing. CD31/α-SMA dual immunofluorescence (Figure 8E) revealed qualitative differences in vascular regeneration: Control sections displayed sparse, faintly stained immature vasculature, indicating a state of early healing and repair. In contrast, the GH group was characterized by features of the inflammatory phase, including a peak infiltration of neutrophils and macrophages, which resulted in higher cellular density. Whereas, the Ca@Q-E@SGH group exhibited mature microvascular networks with intact luminal structures and significantly increased α-SMA^+^ pericyte coverage, demonstrating its progression into the proliferative or remodeling phases. Quantitative analysis confirmed superior CD31 and α-SMA fluorescence intensities in Ca@Q-E@SGH group versus all controls (***P < 0.001), demonstrating effective reversal of diabetes-impaired angiogenesis to restore oxygen/nutrient supply.

## Conclusion

Addressing the critical challenges of persistent inflammation and hypoxic microenvironment in diabetic wound healing, this study innovatively developed an integrated therapeutic strategy combining smart nanosystems with anti-fouling functional hydrogels. The designed Ca@Q-E nanosystem, featuring dual ROS/glucose-responsive characteristics, enables precise EGCG release to simultaneously suppress inflammatory responses and sustainably ameliorate tissue hypoxia. A bilayer hydrogel carrier was further engineered, with its lower layer efficiently loading nanoparticles while the upper layer forms a robust hydrophilic anti-fouling barrier that effectively prevents pathogen invasion. Experimental validation demonstrated that this integrated system (Ca@Q-E@SGH) orchestrates macrophage phenotypic switching from pro-inflammatory M1 to reparative M2 polarization, significantly downregulating pro-inflammatory factors (TNF-α/IL-6) while upregulating IL-10 expression to remodel the wound microenvironment toward an anti-inflammatory state. The Ca@Q-E@SGH system protects extracellular matrix stability by inhibiting MMP-9 over-activation, promotes organized collagen deposition and mature vascular network formation, and accelerates re-epithelialization, ultimately achieving 99.1% wound closure by day 14. This study is currently limited to preclinical animal models. Before clinical translation can be considered, the potential immunogenicity and long-term biosafety of the composite material require further systematic investigation. Furthermore, its scalability and stability under industrial manufacturing conditions have not yet been assessed. This synergistic material design, which integrates intelligent molecular regulation, sustained oxygen supply, and physical anti-fouling functionality, provides a promising strategy for the effective clinical management of diabetic chronic wounds.

## Supplementary Material

rbaf134_Supplementary_Data

## Data Availability

Data will be made available on request.
