# Supplementary material for: Engineered bilayer hydrogel with spatiotemporal drug and oxygen delivery for diabetic wound microenvironment reprogramming
Source: Regen Biomater. 2025 Dec 27;13:rbaf134. doi: 10.1093/rb/rbaf134 (PMC13037811; doi:10.1093/rb/rbaf134)
Supplement: rbaf134_Supplementary_Data [file rbaf134_supplementary_data.zip › Supporting_Information.docx]

**Engineered Bilayer Hydrogel with Spatiotemporal Drug and Oxygen Delivery for Diabetic Wound Microenvironment Reprogramming**

Huaping Li^1, †^, Quan Chen^1, †^, Bihua Liang^1^, Huiyan Deng^1^, Chao Bi^1^, Liqian Peng^1^, Jiaoquan Chen^1^, Shanshan Ou^1^, Luoyu Zhang^1^, Ziyan Chen^1^, Huilan Zhu^1,^ *

^1^Guangzhou Dermatology Hospital, Institute of Dermatology, Guangzhou Medical University, Guangzhou, Guangdong, 510030, China

*Correspondence address. E-mail: zhlhuilan@126.com (Z. H.)

^†^These authors contributed equally to this work.





**Figure S1** FT-IR spectrums of QCS and QCS-PBA.





**Figure S2** Oxygen concentration measured at different time points.





**Figure S3** FT-IR spectrums of Gel and GP.


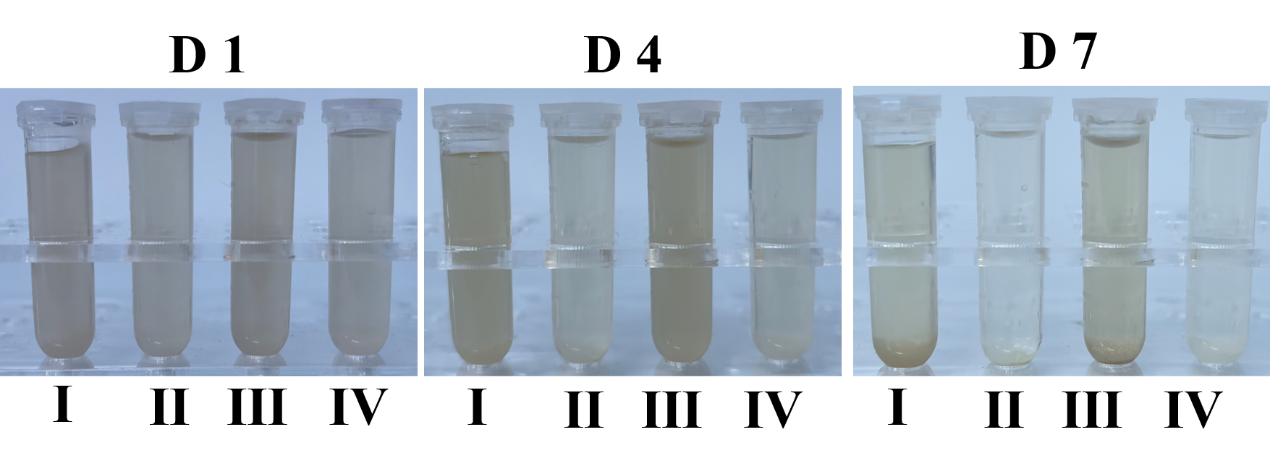


**Figure S4** The physical images of Ca@Q-E nanoparticles after being left in different media for different periods of time. (Ⅰ: PBS, Ⅱ: 10mM H_2_O_2_, Ⅲ: 10mM Glu, Ⅳ: 10mM H_2_O_2_ + 10mM Glu).


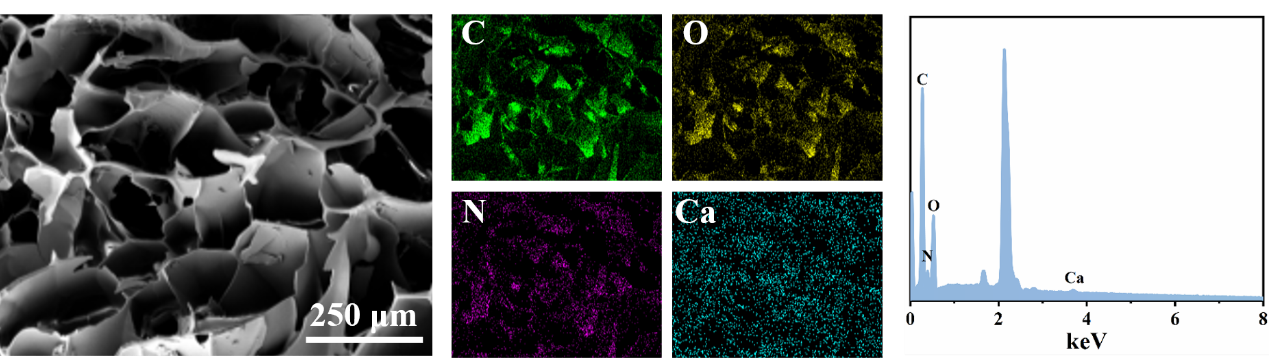


**Figure S5** The SEM image of Ca@Q-E@SGH hydrogel, elemental scanning analysis (C, O, N, Ca) and EDS diagram.


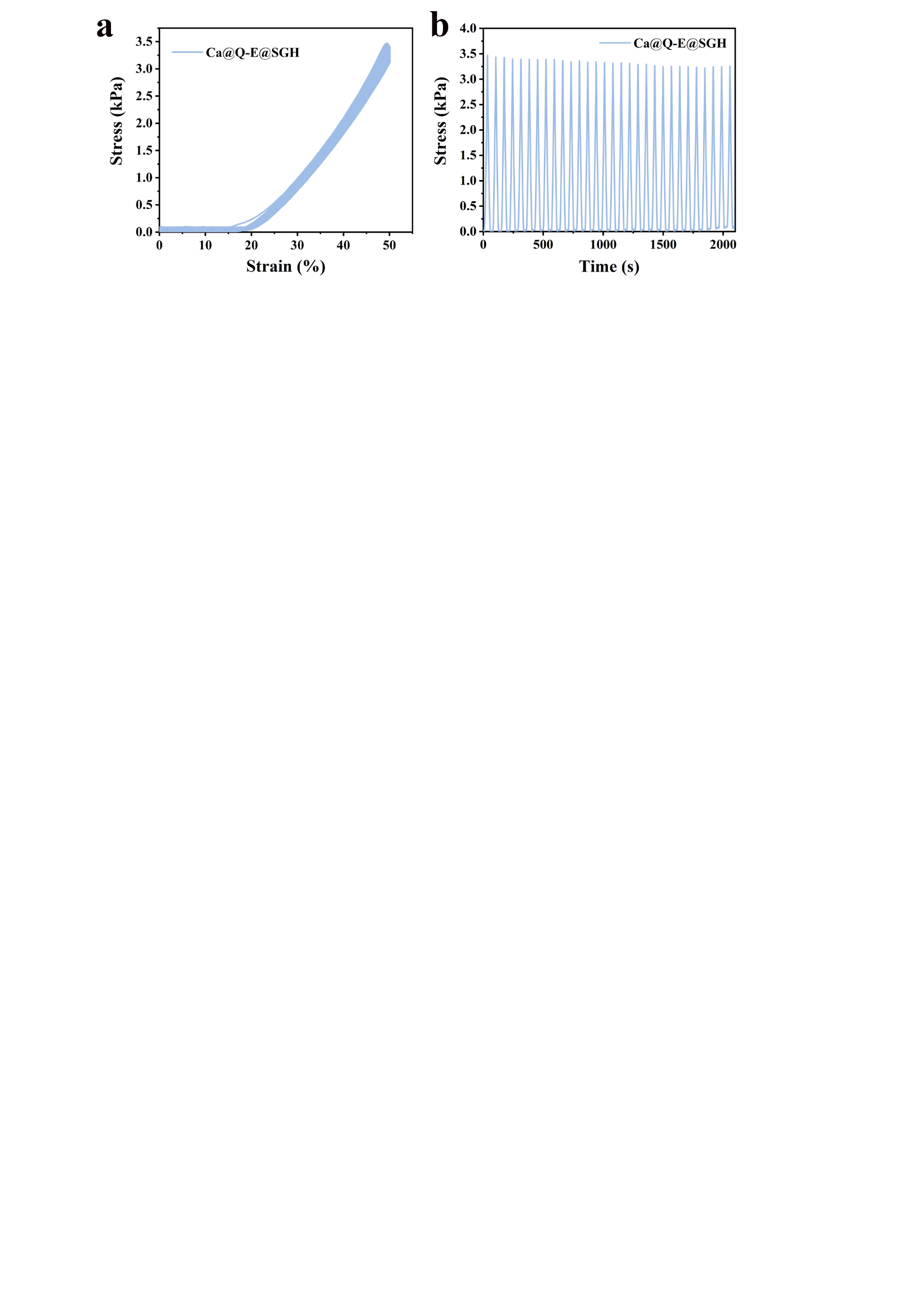


**Figure S6** (a) The stress-strain curve of Ca@Q-E@SGH after 30 cycles of compression and expansion. (b) The time-stress curve of the compression cycle of Ca@Q-E@SGH for 30 times.





**Figure S7** Protein standard curve.


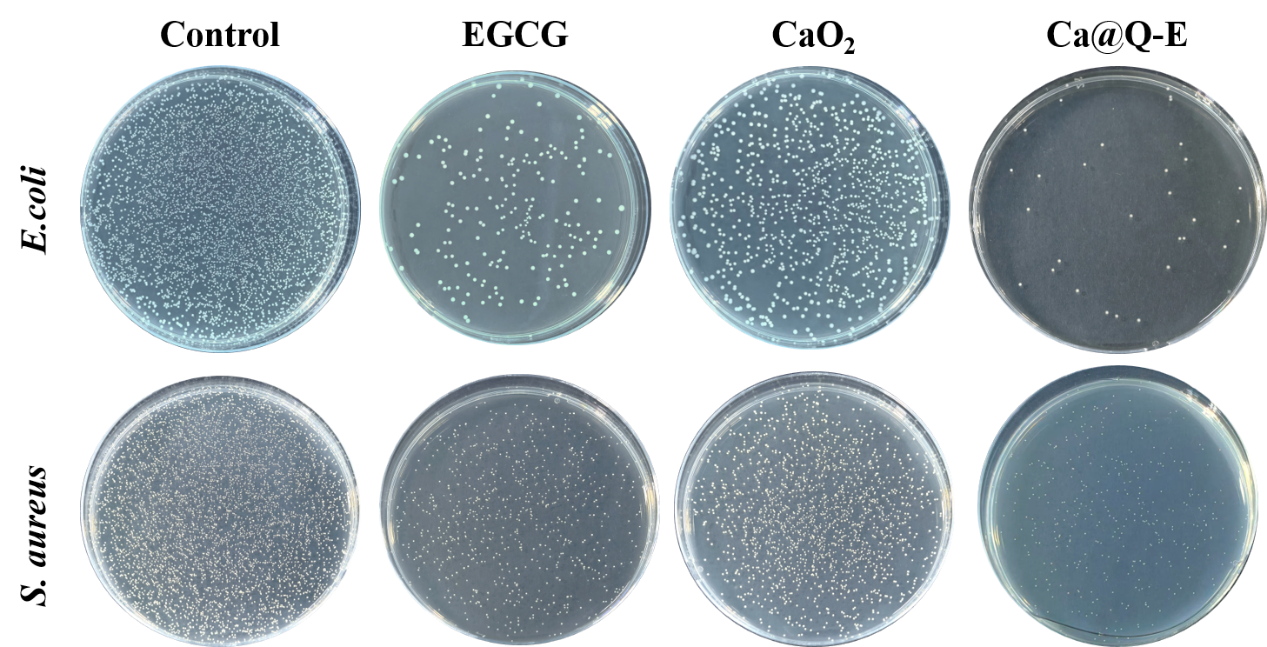


**Figure S8** Representative colony images of *E. coli* and *S. aureus* incubation with PBS, EGCG, CaO_2_ and Ca@Q-E.


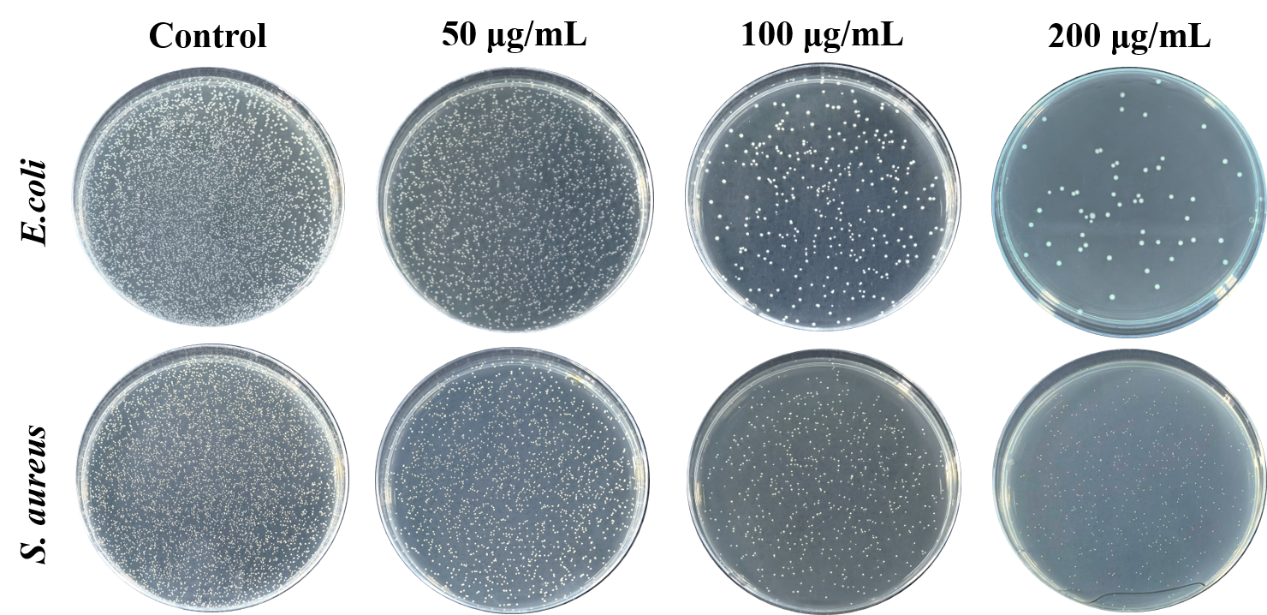


**Figure S9** Representative colony images of *E. coli* and *S. aureus* incubation with different concentrations of Ca@Q-E.





**Figure S10** The scavenging ratios of ABTS free radicals by different components.





**Figure S11** The scavenging ratios of DPPH free radicals by different components.





**Figure S12** Fluorescence intensity of DCFH-DA.





**Figure S13** Fluorescence intensity of HIF-1α.





**Figure S14** Ratio of M1 and M2 phenotypes of macrophages.





**Figure S15** Statistics on the number of hair follicles regenerating on the 14th day after wound repair.





**Figure S16** Statistics on collagen content on the 14th day of wound repair.


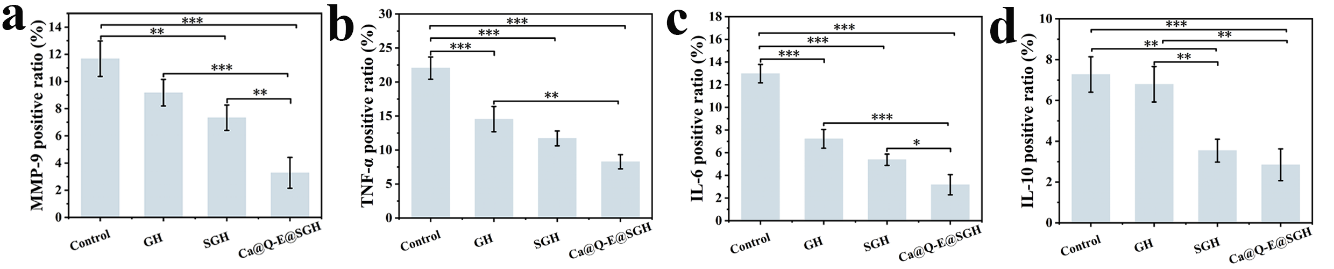


**Figure S17** Quantitative statistics of immunohistochemical positive ratios (a) MMP-9, (b) TNF-α, (c) IL-6 and (d) IL-10.

**Table S1** The Zeta potential of nanoparticles.

| **Group** | **Zeta potential** |
| --- | --- |
| CaO_2_ | -8.50 |
|  | -6.01 |
|  | -3.76 |
| Ca@Q | 7.53 |
|  | 11.52 |
|  | 14.01 |
| Ca@Q-E | -17.25 |
|  | -15.44 |
|  | 13.16 |

**Table S2** Recent comparative studies on the ratio of diabetic wound healing.

| **Author (Year of Publication)** | **Intervening measure** | **Wound healing results** | | |
| --- | --- | --- | --- | --- |
| Wu et al. (2025)^[1]^ | CeO_2_NPs, anchor gallic acid, PTT effect | | 85-90% (12^th^ day) |  |
| Fan et al. (2025)^[2]^ | Ginseng-derived nanoparticles | | 70% (10^th^ day) |  |
| Rui et al. (2025)^[3]^ | Peroxisome proliferator | | 90% (9^th^ day) |  |
| Zhang et al. (2025)^[4]^ | Tannic acid, β-peptide polymer | | 90% (9^th^ day) |  |
| Shao et al. (2025)^[5]^ | Alginate, copper polydopamine, metformin, deferoxamine mesylate | | 92% (14^th^ day) |  |
| Geng et al. (2025)^[6]^ | Protocatechualdehyde, Co | | 90% (14^th^ day) |  |
| Li et al. (2025)^[7]^ | Mn_3_O_4_ nano-enzyme | | 95% (10^th^ day) |  |
| Liu et al. (2025)^[8]^ | Berberine, polydopamine, Zeolitic Imidazolate Framework‐8, PTT effect | | 97% (14^th^ day) |  |
| Deng et al. (2025)^[9]^ | Dual growth factors (GFs) silk sericin hydrogel | | 91% (12^th^ day) |  |
| Wang et al. (2025)^[10]^ | poly-L-lysine, PDA | | 95% (14^th^ day) |  |
| This work | EGCG, CaO_2_, anti-fouling | | 92% (9^th^ day), 99% (14^th^ day) |  |

**References**

1. Wu W, Ye Y, Jiang Q, Zeng K, Wu W, Xie H, Luo T, Guo L. Ligand-metal charge transfer and photothermal carbon dots synergistically enhance cerium dioxide nanozyme activity for diabetic infected wound healing. *Materials Today Bio.* 2025; *35*: 102516.<https://doi.org/10.1016/j.mtbio.2025.102516>

2. Fan L, Jia X, Dong F, Yang S, Li W, Zhao R, Yin L, Zhao D, Wang J. Ginseng-derived nanoparticles accelerate diabetic wound healing by modulating macrophage polarization and restoring endothelial cell function. *Materials Today Bio.* 2025; *34*: 102143.<https://doi.org/10.1016/j.mtbio.2025.102143>

3. Rui S, Xiao F, Li Q, Yang M, Dai L, Yu S, Zhang X, Jiang X, Ahn S, Wang W, Armstrong D G, Wang H, Huang G, Deng W. Peroxisome proliferator-activated receptors-mediated diabetic wound healing regulates endothelial cells’ mitochondrial function via sonic hedgehog signaling. *Burns & Trauma.* 2025; *13*: tkaf063.10.1093/burnst/tkaf063

4. Zhang H, Liang Q, Ji Y, Chen Q, Jiang W, Zhang D, Wu Y, Yu L, Chen W, Liu R. Facile fabrication of antioxidative and antibacterial hydrogel films to accelerate infected diabetic wound healing. *Bioactive Materials.* 2025; *53*: 386-403.<https://doi.org/10.1016/j.bioactmat.2025.07.026>

5. Shao S, Zhu A, Chai Y, Song Z, Chen Y, Xie Y, Lv Y, Huang X, Wang W, Li J, Zhang Q, Kong D, Tan Q. Sequentially triggered triple-responsive hydrogels for targeted regulation of inflammation and angiogenesis in diabetic-infected wound healing. *Nano Research.* 2025; *18*(11): 94907883.10.26599/NR.2025.94907883

6. Geng X, Li W, Qu J, Wang Q, Che T, Yan L, Cui H, Liu D, Qin S. Modified Fish-Skin-Collagen-Based Hydrogels with Antioxidant and Antibacterial Functions for Diabetic Wound Healing. *Advanced Healthcare Materials.* 2025; *14*(21): 2501456.<https://doi.org/10.1002/adhm.202501456>

7. Li Z, Zheng A, Liang C, Mao Z, Deng T, Cao L, Wang C. ROS scavenging Mn3O4 nanozyme regulated immune microenvironment and affects intercellular interaction to promote wound healing in diabetes. *Regenerative Biomaterials.* 2025; *12*: rbaf089.10.1093/rb/rbaf089

8. Liu X, Liu S, Ma B, Zhang Y, Meng Q, Chen M, Chen F, Tian M, Liu S, Liu P, Cai K. A Hydrogel with Low-Temperature Photothermal Therapy and ROS Scavenging Capability for Healing Infected Diabetic Wound. *Advanced Healthcare Materials.* 2025; *n/a*(n/a): e00355.<https://doi.org/10.1002/adhm.202500355>

9. Deng H, Wang F, Zhou Y, Lei H, Zhou H, Chen S, Meng Z, He M, Tu D, Wang H, Li X, Xia Q, Li X, Wang F. Biosynthesis of a dual growth factors (GFs) functionalized silk sericin hydrogel to promote chronic wound healing in diabetic mice. *Bioactive Materials.* 2025; *52*: 511-528.<https://doi.org/10.1016/j.bioactmat.2025.06.017>

10. Wang Z, Sun Z, Zhu S, Qin Z, Yin X, Ding Y, Gao H, Cao X. A multifunctional hydrogel loaded with magnesium-doped bioactive glass-induced vesicle clusters enhances diabetic wound healing by promoting intracellular delivery of extracellular vesicles. *Bioactive Materials.* 2025; *50*: 30-46.<https://doi.org/10.1016/j.bioactmat.2025.03.025>
